# Integrated Full-Length Transcriptome and MicroRNA Sequencing Approaches Provide Insights Into Salt Tolerance in Mangrove (*Sonneratia apetala* Buch.-Ham.)

**DOI:** 10.3389/fgene.2022.932832

**Published:** 2022-07-11

**Authors:** Beibei Chen, Zeyi Ding, Xiang Zhou, Yue Wang, Fei Huang, Jiaxin Sun, Jinhui Chen, Weidong Han

**Affiliations:** ^1^ College of Coastal Agricultural Science, Guangdong Ocean University, Zhanjiang, China; ^2^ Hainan Yazhou Bay Seed Laboratory, Sanya Nanfan Research Institute of Hainan University, Sanya, China

**Keywords:** *Sonneratia* specific, third-generation sequencing, microRNA, target gene, salt stress

## Abstract

MicroRNAs (miRNAs) are small RNA molecules that serve as key players in plant stress responses. Although stress-regulated miRNAs have been explored in various plants, they are not well studied in mangroves. Herein, we combined PacBio isoform sequencing (Iso-Seq) with BGISEQ short-read RNA-seq to probe the role of miRNAs in the salt stress response of the mangrove plant, *Sonneratia apetala* Buch.-Ham. A total of 1,702,463 circular consensus sequencing reads were generated that produced 295,501 nonredundant full-length transcripts from the leaves of a 1-year-old *S. apetala*. After sequencing nine small RNA libraries constructed from control and 1- and 28-day 300 mM NaCl treatments, we identified 143 miRNAs (114 known and 29 novel) from a total of >261 million short reads. With the criteria of |log_2_FC| ≥ 1 and q-value < 0.05, 42 and 70 miRNAs were differentially accumulated after 1- and 28-day salt treatments, respectively. These differential accumulated miRNAs potentially targeted salt-responsive genes encoding transcription factors, ion homeostasis, osmotic protection, and detoxificant-related proteins, reminiscent of their responsibility for salinity adaptation in *S. apetala*. Particularly, 62 miRNAs were *Sonneratia* specific under salt stress, of which 34 were co-expressed with their 131 predicted targets, thus producing 140 miRNA–target interactions. Of these, 82 miRNA-target pairs exhibited negative correlations. Eighteen miRNA targets were categorized for the ‘environmental information processing’ during KEGG analysis and were related to plant hormone signal transduction (ko04075), MAPK signaling pathway–plant (ko04016), and ABC transporters (ko02010). These results underscored miRNAs as possible contributors to mangrove success in severe environments and offer insights into an miRNA-mediated regulatory mechanism of salt response in *S. apetala*.

## Introduction

Soil salinity, one of the major environmental threats, negatively affects plant growth and development. Worldwide, approximately 20% of irrigated soils and 50% of arable land are exposed to salt stress (Syed et al., 2021). High salinity also leads to secondary stresses such as oxidative stress and nutritional imbalance that result in cellular damage, growth inhibition, and yield decrease. Generally, genetically engineering plants to increase salt tolerance would be a promising approach. Thus, deciphering the molecular mechanism and physiological processes that are involved in response to salt stress would certainly facilitate the identification of candidates for genetic engineering of stress-resistant plants, and could help in coping with stress challenges.

Mangroves are a group of halophytic woody plants, growing in tropical and sub-tropical estuaries and intertidal zones, which play a vital role in water purification, coastal protection and the maintenance of biodiversity (Wang and Gu, 2021). As extremophiles, mangroves have evolutionarily adapted to tolerate high temperatures, flooding, anoxia, and high salt conditions in typically nutrient-poor environments. With continued exposure to highly salinized soil and periodic seawater erosion, salt-tolerance mechanism of mangroves especially differs from that of terrestrial freshwater plants. So, there has been a growing interest in exploring the mechanism of salt stress adaptations of mangroves. Generally, mangroves exhibit several physiological strategies for handling salt, which include salt excretion (e.g., *Sonneratia*, *Aegicaras*, *Avicennia*), salt balance modulation (e.g., *Xylocarpus*, *Bruguiera*, *Rhizophora*), and hyperexclusion (e.g., *Heritiera*) (Dassanayake et al., 2009). Many studies have been devoted to uncovering genetic regulators that modulate mangroves’ adaptations to salt environments. Previous research (Srikanth et al., 2016) indicated that fructose-1,6-bisphosphate (FBP) osmotin and aldolase were related to salt resistance in *B. gymnorrhiza* roots. A *vacuolar Na* ^*+*^ */H* ^*+*^ *antiporter* served as a crucial actor in cellular salinity adjustments of *R. apiculata* (Saddhe and Kumar, 2019). The mRNA expressions of catalase, Cu–Zn SOD, and ferritin genes have been studied in response to saline stress and their role in oxidative stress response has been confirmed in *A. marina* (Dahibhate et al., 2020).

MicroRNAs (miRNAs) comprise a class of endogenous small non-coding RNA molecules that are approximately 21 nt in length, and are recognized as important regulators of the transcriptional and post-transcriptional expression of genes, mediated via mRNA degradation, transcription inhibition or the repression of mRNA translation (Tyagi et al., 2019). Besides their roles in modulating diverse essential physiological, biochemical and molecular processes, many miRNAs were discovered to respond to various abiotic stimuli in plants, including drought (Li et al., 2021), salinity (Hu et al., 2018; He et al., 2021; Salgado et al., 2021), cold (Hu et al., 2018) and oxidative stress (Li et al., 2020), as well biotic stresses (Salamon et al., 2021). To address salinity-induced stress, multiple gene expression mechanisms have evolved in plants, which relate to a wide range of biological processes, including transcription, signal transduction, energy metabolism, membrane trafficking, protein biosynthesis, and photosynthesis (Pan et al., 2018). Understanding of miRNA-guided biological regulations in plants against salinity could provide the necessary basis for unraveling the complex molecular and genetic mechanism underlying salt-stress resistance. A growing body of evidences has demonstrated that miRNA-mediated gene regulation is indispensable for plants response to salt stress**.** For instance, miR172c regulates root plastic development in soybean response to salt by targeting *NNC1* (Gupta et al., 2021); the miR398-*CSD* module protects cell membrane structure via detoxifying superoxide radicals against salt stress in tomato (Gao et al., 2022), miR414/miR408/miR164e could modulate gene recombination replication, thereby repairing to resist saline environment (Yang et al., 2020). Despite their critical roles of in-plant tress resistance, miRNAs have been studied in only a few woody plant species, such as poplar (Si et al., 2014), paulownia (Zhao et al., 2018), and willow trees (Zhou J. et al., 2012). In particular, little research has been performed on mangrove miRNAs. Currently, the miRNA repertoires have only been reported for few mangrove species, including *Avicennia marina* (Forsk.) Vierh. (Khraiwesh et al., 2013), *Bruguiera gymnorhiza* (L.) Lam. (Wen et al., 2016; Dasgupta et al., 2017), *Kandelia candel* (Linn.) Druce (Wen et al., 2016), *Rhizophora apiculata* Bl. (Singh et al., 2020) and *Sonneratia alba* Sm. (Wang et al., 2021a). Limited attempt has been made to dissect the molecular basis of salt stress adaptation that is guided by miRNAs (Khraiwesh et al., 2013; Wen et al., 2016).

To detect stress-related miRNAs, it is necessary to compare expression patterns of miRNAs in plants under normal and stress-treated growth conditions. With the implementation of next-generation sequencing, it became easier and cost-efficient to carry out genome-wide mining and identification of stress-responsive miRNAs. However, high throughput sequencing produces short reads and thereby acquires incomplete assembly of sequences. On the other hand, the third-generation long-read sequencing platforms offer better completeness in full-length cDNA sequencing and yield more accurate isoform-level transcripts. Indeed, third-generation PacBio sequencing technology (Iso-Seq) has been successfully harnessed in multiple species and has revealed greater accuracy in exploring genome information (Pearman et al., 2020; Sharma et al., 2021). The combination of second- and third-generation platforms has become an effective approach for critical gene identification and function mining, especially for those with no sequenced genomes available (Mei et al., 2021; Meng et al., 2021; Wang et al., 2021b). In this study, we jointly employed short-read RNA-seq and PacBio Iso-Seq to produce a high-confidence full-length transcriptome dataset of 1-year-old *S. apetala* individuals and further used them to identify salt-responsive miRNAs through constructing small RNA libraries from the leaves of *S. apetala* seedlings treated with 300 mM NaCl for 0 days (control), 1, and 28 days. To study the genetic mechanism of the miRNA-mediated gene modulation in salt stress adaptations, the potential targets of salt-related miRNAs were predicted and further analyzed. Most importantly, *Sonneratia*-specific miRNAs were screened, with the co-expressed miRNA-target regulatory interactions were investigated using transcriptome data, as well as Gene Ontology (GO) and KEGG analyses. In particular, we focused on negatively correlated *Sonneratia*-specific miRNA-target pairs and the underlying mechanism in salinity response. This study provided systematically characterized salt-related miRNAs of *S. apetala* and the expanded features of putative targets reveal the miRNA inferred regulatory networks responding to excessive saline stress. This would be helpful for further investigation of molecule functions during the salt response in mangroves.

## Materials and Methods

### Plant Materials and Salt Treatments

The seeds of *S. apetala* were collected from adult plants growing along a mangrove coastal belt in Techeng Island (21°09′∼21°10′ N, 110°25′∼110°27′ E), Guangdong, China and were sown on artificial soil in seedbeds. After approximately 70 days, seedlings with 10–16 cm in height were transplanted into polythene bags and watered every 2 days. For the salt stress treatment, 1-year-old uniformly developed seedlings were selected and salinity stress was induced by adding the salt to the Hoagland’s solution. Plants grown on sandy soil were watered with 300 mmol/L NaCl, and the controls with only water. A time-course was used for deep sequencing (0, 1, and 28 days) and named LCK, LT1, and LT2, respectively. Three biological replicates were used for each time point and leaf samples were collected from the nine plants (LCK_a, LCK_b, LCK_c, LT1_a, LT1_b, LT1_c, LT2_a, LT2_b, and LT2_c) for RNA extraction. The collected tissues were immediately frozen in liquid nitrogen and then kept at -80°C for RNA extraction until further processing.

### PacBio Iso-Seq Library Preparation and Sequencing

Total RNA was extracted from each sample using an RNeasy Plant mini kit (Qiagen, Hilden, Germany) according to the manufacturer’s protocol. The quality and integrity of RNAs were measured by Nanodrop 2000 (Thermo Scientific) and Agilent 2100 Bioanalyzer (Agilent Technologies, CA, United States). Only RNA samples with 1.8 < OD260/280 < 2.2 and RIN ≥7.0 (RNA Integrity Number) were used for follow-up experiments. The qualified RNA samples were used for further PacBio and BGISEQ library construction, respectively.

For PacBio sequencing library construction, we combined equal amounts of total RNA from the nine replicates, as well as total RNA extracted from root tissues of *S. apetala* after salt treatment for 0, 7 days (200 and 400 mmol/L NaCl), and 14 days (300 mmol/L NaCl). The mixed RNA sample was reverse-transcribed into cDNA using the SMARTer™ PCR cDNA Synthesis Kit. PCR amplification was performed to amplify the cDNAs and the fragments were then subjected to damage repair, end repair, and connect SMRT dumbbell-shaped adapters for a full-length transcriptome library construction. The concentration of SMRTbell library was assessed using a Qubit 3.0 fluorometer with a Qubit™ 1X dsDNA HS Assay kit (Invitrogen, Carlsbad, United States) and the quantified criteria for the library quality was a concentration of >10 ng/μl. Then the qualified full cDNA library was sequenced using the binding kit 2.1 from PacBio Sequel platform at the Beijing Genomics Institute (BGI), China. The raw Iso-Seq data were processed to obtain subread sequences *via* SMRTlink v6.0 software and short-read sequences were finally used for calibration of the consensus sequence to obtain a high-quality sequence.

### Transcriptome Sequencing and Differentially Expressed Unigene Identification

After the library was constructed following the methods described by Li et al. (2019), transcriptome sequencing was performed using the BGISEQ-500 sequencing platform with paired-end sequencing (length of 150 bp). Raw reads were filtered by the removal of low-quality sequence fragments, reads containing N blurs, and adapter sequences. The Trinity software (v2.0.6) with default parameters and a minimum contig length of 150 bp was used for assembly generation. Transcript levels were determined from the short-read data through RSEM (Li and Dewey, 2011), with the resulting full-length transcripts used as a reference sequence (ref). The transcript isoform level and gene-level counts were converted into fragments per kilobase of transcript per million mapped reads (FPKM) values. Analysis of differential expression of genes between different treatments was performed using the DESeq R package (1.10.1) with DEGs selected using log2 FC ≥ 1 or ≤ −1, *p* < 0.01 and Q < 0.05.

### Small RNA Isolation and BGISEQ Sequencing

Small RNA libraries for the nine individuals of *S. apetala* were constructed according to a previously described procedure (Fehlmann et al., 2016). Briefly, small RNA fragments with a length of 10–30 nt were purified by using a 15% urea-PAGE gel, followed by ligating with adenylated 3′ and 5′ adapters. Then, Adapter-ligated RNAs were reverse-transcribed and the cDNA product was amplified by PCR. The concentration and purity of the PCR yield were quantified by Qubit 2.0 (Invitrogen, Cat No. Q33216). Finally, approximately 20 μg product for each sample was sequenced on a BGISEQ-500 platform, and 50 bp single-end reads were generated.

### Identification of Conserved and Novel MicroRNAs

Clean sequencing reads were obtained after discarding the contaminations and low quantity reads. AASRA (Tang et al., 2021) and CMsearch (Nawrocki and Eddy, 2013) were used to map clean reads to the full-length transcript reference sequences and other sRNA databases. To ensure that each unique small RNA was mapped to only one category, we followed a priority rule: miRBase > piRNABank > snoRNA (human/plant) > Rfam > other sRNA. Novel miRNAs were predicted by miRA (Evers et al., 2015) software by evaluating the characteristics of hairpin structures of miRNA precursors with the assistance of RNAfold web server (http://www.tbi.univie.ac.at/RNA/) (Lorenz et al., 2011).

### Abundance Analyses and Target Prediction of MicroRNAs

The abundance of miRNA transcripts was normalized using the transcripts per million (TPM). Differentially accumulating miRNAs for each pair of stress treatments (LT1 vs*.* LCK and LT2 vs*.* LCK) were identified *via* DESeq2 (Love et al., 2014) and based on the normalized TPM counts. The settings “|log2Fold change. normalized| ≥ 1” with “*p* < 0.01” and “Q < 0.05” were used as thresholds for determining significant changes in transcript abundances. To increase the accuracy of the results, two types of software, psRobot (Wu et al., 2012) and TargetFinder (Fahlgren and Carrington, 2010), were used for target gene prediction and the intersection of the target gene was chosen as the final results. Functional enrichment analysis for miRNA target genes was performed through screening the GO (www.geneontology.org/) and KEGG Pathway databases (www.genome.jp/kegg/) with “Q < 0.1” as a threshold to determine significant enrichments.

### Quantification of MicroRNA Transcript Levels *via* Real-Time Quantitative Polymerase Chain Reaction

cDNAs were synthesized by reverse transcription of total RNA from nine *S. apetala* samples (LCK_a, LCK_b, LCK_c, LT1_a, LT1_b, LT1_c, LT2_a, LT2_b, and LT2_c). RT-qPCRs were carried out on a CFX Connect™ Real-Time PCR Detection System using SYBR green-based real-time PCR with miRNA-specific forward primer and universal reverse primer (Supplementary Table S1). The PCR program included an initial denaturation at 94°C for 3 min, and 40 cycles of 20 s at 94°C, and 60°C for 40 s. The specificity of the amplified fragments was checked using the generated melting curve and the 2^–△△Ct^ method was used to calculate the abundance of each miRNA against U6 gene (Khraiwesh et al., 2013). All RT-qPCR amplifications were performed in triplicate (technical repetitions) to ensure reproducibility and reliability.

## Results

### Sequencing and Data Analysis

In this study, the full-length transcriptome of *S. apetala* was sequenced using PacBio isoform sequencing (Iso-Seq) of a pooled RNA sample. A total of 1,702,463 long reads were generated that produced 295,501 unique transcript isoforms with a total length of 418,900,351 base pairs (bp). This formed the *S. apetala* reference sequence database for the identification of known and novel miRNAs in *S. apetala*, as well as for the prediction of corresponding miRNA-target genes.

Nine libraries were constructed for high-throughput small RNA (sRNA) sequencing from NaCl-free (LCK) and NaCl-treated (1 d and 28 days) *S. apetala* leaves. Each library yielded more than 29 million raw sRNA reads (Table 1). After trimming adapters contaminants, low-quality tags, RNAs shorter than 18 nt, poly (A) sequences, and other artifacts, 27,207,049 (LCK_a), 27,039,294 (LCK_b), 26,410,458 (LCK_c), 26,298,084 (LT1_a), 26,776,814 (LT1_b), 26,685,055 (LT1_c), 26407348 (LT2_a), 25589700 (LT2_b) and 26356554 (LT2_c) clean reads were acquired for further analysis (Table 1). Filtered reads were aligned and mapped to the *S. apetala* full-length transcriptome sequence and the mapping rate for each library varied from 80.83% to 90.95% (Table 2). In addition, Pearson’s correlation coefficient was calculated based on the abundance of sRNAs, which accounted for a minimum value of 0.889 between samples (Supplementary Figure S1). These results indicated the good quality of sequencing data that could be used for the follow-up analyses.

**TABLE 1:** Summary of sequencing data for each sample.

Sample name	Raw tag count	Low-quality tag count	Invalid adapter tag count	PolyA tag count	Short valid length tag	Clean tag count	Q20 of clean tag (%)	Percentage (%)
LCK_a	32000000	253912	438472	193	4100374	27207049	98.9	85.02
LCK_b	31578947	277052	411545	184	3850872	27039294	99	85.62
LCK_c	31168831	258730	436685	374	4062584	26410458	99.1	84.73
LT1_a	29721273	345190	588245	896	2488858	26298084	98.9	88.48
LT1_b	30155684	339962	599595	913	2438400	26776814	98.9	88.8
LT1_c	29906976	336268	574248	944	2310461	26685055	98.8	89.23
LT2_a	31802816	300631	790887	1598	4302352	26407348	98.8	83.03
LT2_b	30868831	320640	621507	844	4336140	25589700	99	82.9
LT2_c	31578947	328989	650973	746	4241685	26356554	99	83.46

**TABLE 2:** Annotations of sRNAs perfectly matching *S. apetala* mRNA transcriptome^a^.

Class	0 days			1 days			28 days		
	LCK_a (%)	LCK_b (%)	LCK_c (%)	LT1_a (%)	LT1_b (%)	LT1_c (%)	LT2_a (%)	LT2_b (%)	LT2_c (%)
Clean reads	27207049 (100)	27039294 (100)	26410458 (100)	26298084 (100)	26776814 (100)	26685055 (100)	26407348 (100)	25589700 (100)	26356554 (100)
Total match	23979516 (88.14)	23680675 (87.58)	23064967 (87.33)	21489673 (81.72)	21729758 (81.15)	21570544 (80.83)	23518404 (89.06)	23273943 (90.95)	23753950 (90.13)
Mature (miRNA)	2749347 (10.11)	2602606 (9.63)	2608999 (9.88)	2724558 (10.36)	2811601 (10.50)	2799408 (10.49)	3163093 (11.98)	3125895 (12.22)	3239088 (12.29)
rRNA	834264 (3.07)	833305 (3.08)	857541 (3.25)	672078 (2.56)	691599 (2.58)	682402 (2.56)	669819 (2.54)	670609 (2.62)	727861 (2.76)
snoRNA	536234 (1.97)	533179 (1.97)	522190 (1.98)	470373 (1.79)	476742 (1.78)	469302 (1.76)	408215 (1.55)	398682 (1.56)	418031 (1.59)
snRNA	530600 (1.95)	527374 (1.95)	518043 (1.96)	461389 (1.75)	468199 (1.75)	469981 (1.76)	400266 (1.52)	389494 (1.52)	408576 (1.55)
tRNA	533604 (1.96)	530291 (1.96)	505978 (1.92)	457816 (1.74)	465205 (1.74)	465672 (1.75)	401504 (1.52)	392426 (1.53)	412792 (1.57)
Rfam other sncRNA	559549 (2.06)	557155 (2.06)	552662 (2.09)	480907 (1.83)	490443 (1.83)	482088 (1.81)	432887 (1.64)	432974 (1.69)	446797 (1.70)
Unannotated	21463451 (78.88)	21455384 (79.35)	20845045 (78.92)	21030963 (79.97)	21373025 (79.82)	21316202 (79.87)	20931564 (79.25)	20179620 (78.86)	20703409 (78.54)

a The number represented the abundance of the reads generated directly from deep sequencing; 0 day, 1 day, and 28 days represent leaf treated with 300 mM NaCl for 0, 1, and 28 days, respectively.

**FIGURE 1:**
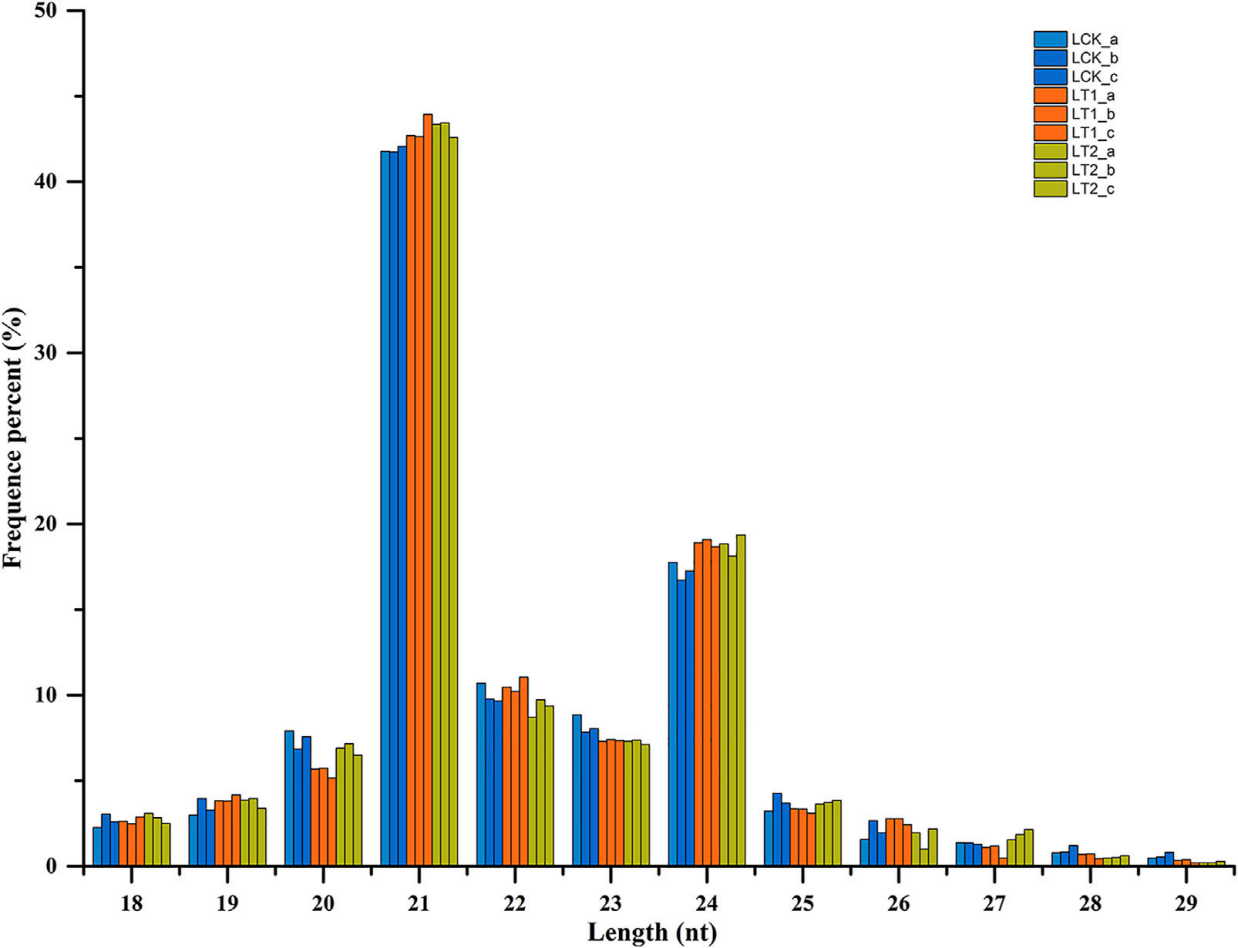
Size distribution of small RNA sequences in different libraries. nt, nucleotides.

The sRNA size distribution in each library is summarized in Figure 1. The most abundant sRNAs ranged from 20 nt to 24 nt in length, which was the typical size range for Dicer-derived products (Wang et al., 2019). For all the nine libraries, 21 nt sRNAs represented the most frequent length, which coincided with the profiles described in other plant species including Populus (Cui et al., 2019), Trifoliate orange (Hu et al., 2022), and grapevine (Chen et al., 2019). The second most abundant class was of 24 nt sRNAs, making up an average of approximately 18.3% of all the sRNAs in the nine libraries (Figure 1). The sRNAs were annotated into several different categories (Table 2). Of these, an average of 2,653,651 (9.87%), 2,778,522 (10.45%), and 3,176,025 (12.16%) unique sRNAs were annotated as miRNAs in LCK, LT1 and LT2 libraries, respectively. All the libraries showed similar distribution patterns for other RNA families (Table 2), including rRNA (∼2.78%), snoRNA (∼1.77%), snRNA (∼1.75%), tRNA (∼1.74%) and Rfam other sncRNA (∼1.85%) (Table 2). Notably, a predominant proportion of sRNA sequences (> 78.54%) were unannotated sRNAs, raising the possibility of the existence of novel miRNAs in *S. apetala* genome.

### Identification of Conserved MicroRNAs in *Sonneratia apetala*

**FIGURE 2:**
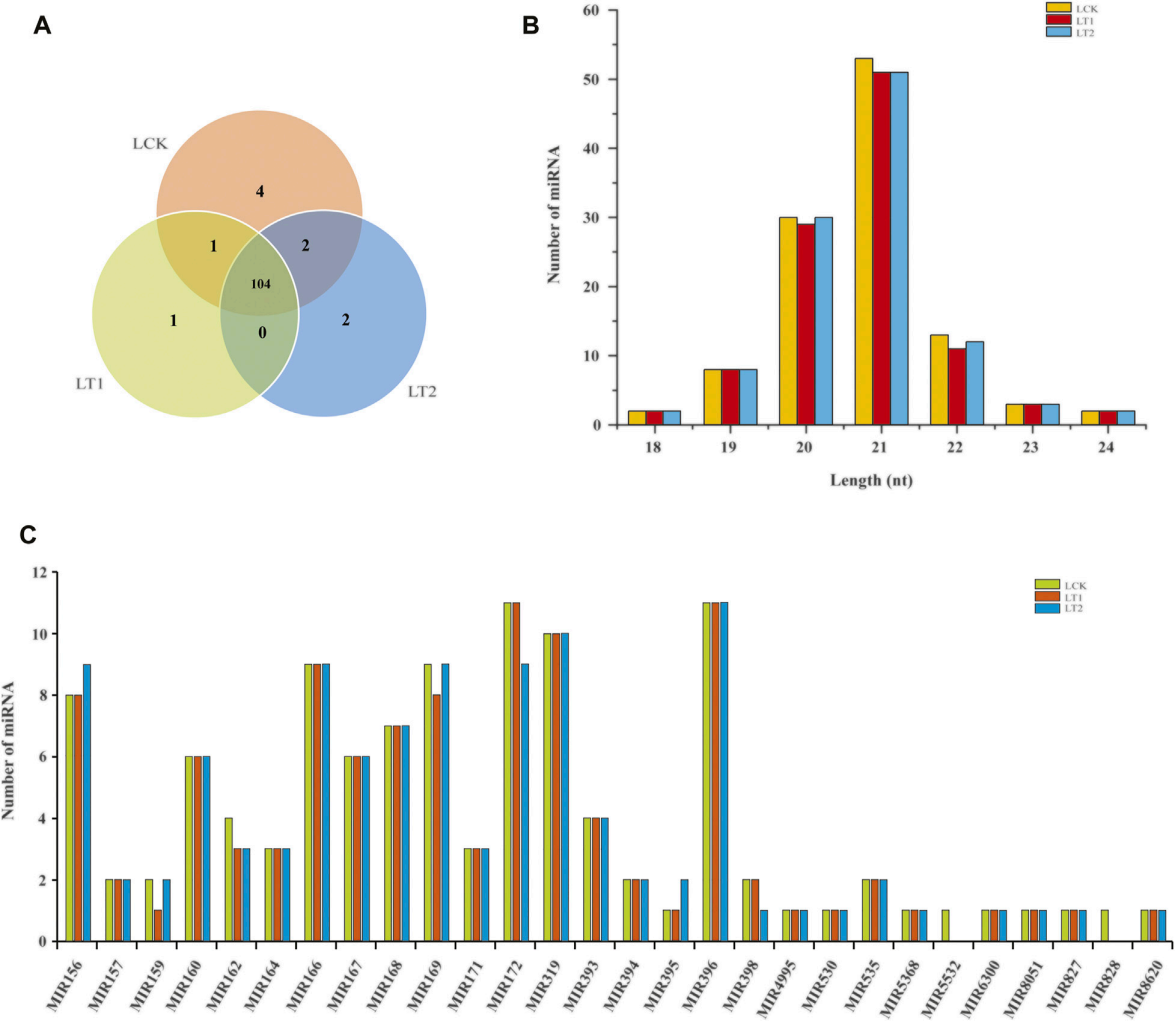
Summary of known miRNAs identified from *S. apetala*. **(A)** Distribution of known miRNAs in samples treated with salt for 0 days (LCK), 1 day (LT1) and 28 days (LT2). **(B)** Size distribution of known miRNAs in LCK, LT1 and LT2 libraries. **(C)** Distribution of known miRNA family size in *S. apetala*.

To identify the miRNAs conserved in *S. apetala*, we aligned the unique sRNA candidates with all the plant miRNAs in the miRBase 22.1 database. Finally, a total of 114 known mature miRNAs, representing 28 miRNA families were identified in *S. apetala*, which were generated from 97 pre-miRNAs (Supplementary Data S1). Of these, 111 conserved miRNAs were found in the control, whereas 106 and 108 miRNAs were found after salt treatment for 1 and 28 days, respectively (Figure 2A; Supplementary Data S1). The length of known miRNAs varied from 18 nt to 24 nt, with 21 nt miRNAs being the most abundant (Figure 2). The next abundant class was 20-nt-long miRNAs, which was different from the total sRNA population (Figures 1, 2B), suggesting the existence of many other types of sRNAs within the libraries. The diversity of conserved *S. apetala* miRNA families could be determined by the number of their members. For example, miR160 and miR166 families comprised 6 and 9 members, respectively. Additionally, most of the conserved miRNA families contained more than one member; the two largest families were miR172 and miR396 with 11 miRNA members, followed by miR319 with nine members. However, some conserved miRNA families, such as miR4995, miR530, miR5368, miR5532, miR6300, miR8051, miR827, miR828, and miR8620, had just one member. The number of members for each family is summarized in Figure 2C.

### Identification of Novel MicroRNAs in *Sonneratia apetala*

**FIGURE 3:**
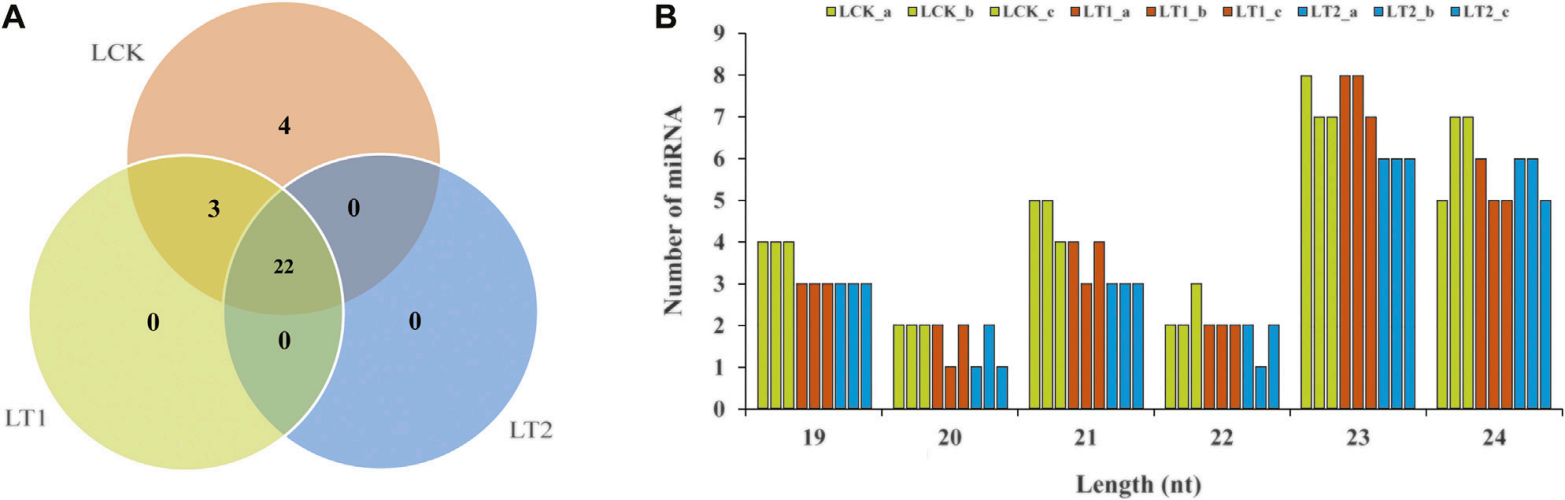
Novel miRNAs identified in *S. apetala* by high-throughput sequencing. **(A)** Distribution of novel miRNAs in control (LCK), 1 day (LT1) and 28 days (LT2) salinity treatment. **(B)** The length distribution of *S. apetala* novel miRNAs. a, b and c represent the three biological repetitions for each treatment.

Among the remaining unannotated sRNAs, 29 were predicted as putative novel miRNAs in *S. apetala* (Figure 3A; Supplementary Data S2). The length of the novel mature miRNAs varied from 19 nt to 24 nt, which was consistent with the size of miRNA fragments produced by AGO-guided cleavage (Yang et al., 2020). Of the newly predicted miRNAs, 23 nt and 24 nt accounted for the two major class sizes (Figure 3B). Not all of the novel miRNAs were detected in all libraries. Four novel miRNAs (Sap-nmiR4, Sap-nmiR9, Sap-nmiR13, and Sap-nmiR15) were uniquely detected in LCK library, whereas three novel miRNAs, namely, Sap-nmiR11, Sap-nmiR12 and Sap-nmiR14, were found in the LCK and LT1 libraries. Moreover, most novel miRNAs (22) were co-expressed in all three different groups (LCK, LT1, and LT2), accounting for 75.9% of the total. Notably, the abundance of these new miRNAs was relatively low (Supplementary Data S2), which was a generic feature of species-specific miRNAs (Feng et al., 2015). The precursor of the 29 potential novel miRNAs ranged in length from 65-nt to 284-nt long, which was concordant with the general length of pre-miRNAs (Lertampaiporn et al., 2013). Furthermore, secondary stem-loop structure of the new miRNAs precursors was also assessed, and their free energies of the thermodynamic ensemble varied from −26.02 kcal/mol to −180.32 kcal/mol with an average value of −80.36 kcal/mol (Supplementary Figure S2; Supplementary File S2). miRNA star sequences (miRNA*) were detected for each novel miRNA (Supplementary Data S2), confirming their authenticity as novel miRNAs.

### Accumulation Profiles of Conserved and Novel MicroRNAs

**FIGURE 4:**
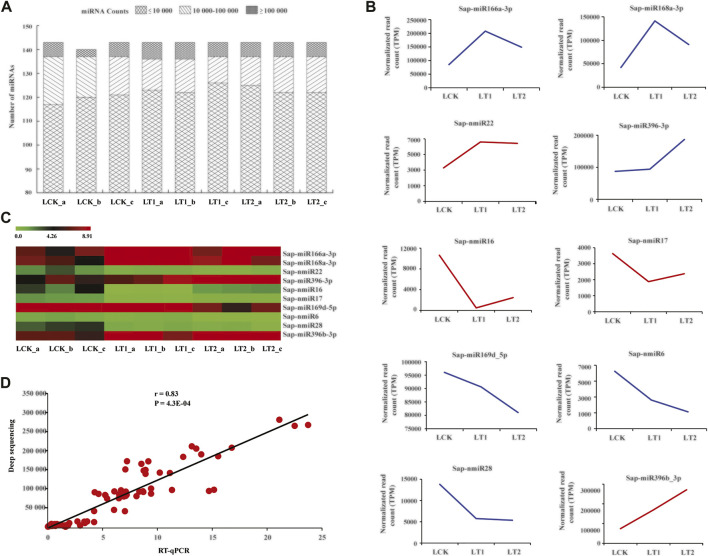
Abundance characteristics of miRNAs obtained from *S. apetala* samples subjected to deep sequencing. **(A)** A global view of miRNA accumulation levels. **(B)** Representative graphs of accumulation profile for 10 most abundant miRNAs. These miRNAs were divided into six categories on the basis of their accumulation patterns, which were distinguished by the colors of lines. **(C)** Relative transcript abundance of the 10 most highly accumulated miRNAs with the help of real-time quantitative polymerase chain reaction (RT-qPCR) in control (LCK), 1 day (LT1) and 28 days (LT2) salinity treatment of *S. apetala*. a, b and c represent the three biological repetitions for each treatment. **(D)** Pearson correlation coefficient (*r*) analysis between accumulation levels by RT-qPCR and deep sequencing for the 10 most abundant miRNAs.

High-throughput sequencing detects the type and abundance of miRNAs (Yang et al., 2020). In this study, miRNA exhibited variable abundances in the nine libraries, with the number of reads ranging from zero to hundreds of thousands, and was exploited as the indicator for assessment of a miRNA’s accumulation level. Sap-miR166a-3p represented the highest abundance with an average of 74,614, 169,800 and 271,226 reads in LCK, LT1 and LT2 libraries, independently (Supplementary Datas S1, S2). Some miRNAs, such as Sap-miR167d_1 and Sap-miR166d-5p, also exhibited extraordinarily high abundances in the three groups, while other miRNAs, including Sap-miR535a, Sap-nmiR16, and Sap-nmiR28, were moderately accumulated, with total reads varying from 10,080 to 97,452 (Figure 4A; Supplementary Data S1). Nevertheless, several miRNAs (Sap-miR164b, Sap-miR396b, Sap-nmiR13, Sap-nmiR26, etc) lad extremely low abundances in all the libraries. Additionally, some miRNA counts diverged sharply across control and treatments. For instance, Sap-miR396a-3p_1 had 6,852 reads in the LCK library and 3,520 reads in the LT1 library, while there were only five reads in the LT2 library. Interestingly, the expression of Sap-nmiR20 and Sap-nmiR24 was similar in all the conditions (control and salinity treatments), with a relatively low accumulation level of 455 and 489 reads per million (TPM). Due to the distinction in their abundances among different libraries, we considered that the candidate miRNAs would have diverse functions in response to salt stress in *S. apetala*.

Thereafter, we selected the 10 most abundant miRNAs (5 conserved and 5 novel) in the three groups and analyzed their patterns of accumulation across the control and two salt treatments: these miRNAs clustered into six groups (Figure 4B). The first cluster contained two miRNAs, Sap-miR166a-3p and Sap-miR168a-3p, which were quickly induced at 1 day of stress and repressed at 28 days; the second group comprised one miRNA, Sap-nmiR22, which was up-regulated at 1 day and then accumulated at a relatively stable level, whereas Sap-miR396-3p in the third group exhibited an opposite pattern; the fourth class, which included Sap-nmiR16 and Sap-nmiR17, was rapidly repressed at 1 day, then up-regulated at 28 days. Finally, Sap-miR169d-5p, Sap-nmiR6, and Sap-nmiR28 in the fifth group were repressed at 1 and 28 days, while Sap-miR396b-3p, in the sixth, showed a specific expression profile being up-regulated under the salt treatment. These findings facilitated our comprehension of the modulation of miRNAs in response to salt stress. We examined the transcript levels of these miRNAs with the help of RT-qPCR in leaf tissues of *S. apetala* that were treated with salt for 0, 1, and 28 days. It revealed a concordance with the expression values from high-throughput sequencing (*r* = 0.83, *p* = 4.3E-04; Figures 4C,D).

### Differential Expression Analysis and Target Gene Prediction of MicroRNAs Responsible to Salt Stress

To investigate the effect of salinity on *S. apetala* miRNAs, we conducted a differential analysis of accumulations between the libraries treated (LT1 or LT2) and non-treated (LCK) with salt. Expression profiles of miRNAs in two comparisons (LT1 vs*.* LCK and LT2 vs*.* LCK) were compared; miRNAs with log_2_-fold changes beyond 1.0 or −1.0, and Q < 0.05 were considered to be differentially accumulated. In total, 73 miRNAs (56 known and 17 novel) were significantly differentially accumulated in response to salt (Supplementary Data S3). Of these, three novel miRNAs, Sap-nmiR9, Sap-nmiR12, and Sap-nmiR14 were markedly differentially accumulated with an absolute value of log_2_-ratio (LT/LCK) > 6. Increases in salt treatment time dramatically increased the number of differentially accumulated miRNAs, from 42 (LT1 vs*.* LCK) to 70 (LT2 vs*.* LCK) in *S. apetala* leaves. However, only 39 salt stress-regulated miRNAs appeared in both two time-points as compared with the control group. Particularly, 3 and 31 miRNAs were especially significantly altered in samples treated with salt for 1 and 28 days, respectively. Additionally, the number of down-regulated miRNAs varied through the time-course compared to that of up-regulated ones (Supplementary Data S3). In LT1 vs*.* LCK comparison, 18 miRNAs (14 known and 4 novel) were up-regulated, whereas 24 miRNAs (17 known and 7 novel) were downregulated by salt stress. In LT2 vs*.* LCK comparison, 36 miRNAs (30 known and 6 novel ones) were induced, while 34 miRNAs (23 known and 11 novel ones) were repressed. Among all the differentially accumulated miRNAs, 38 miRNAs (34 known and 4 novel) responded to stress of salinity in a similar way at different time-points during salt treatment.

**FIGURE 5:**
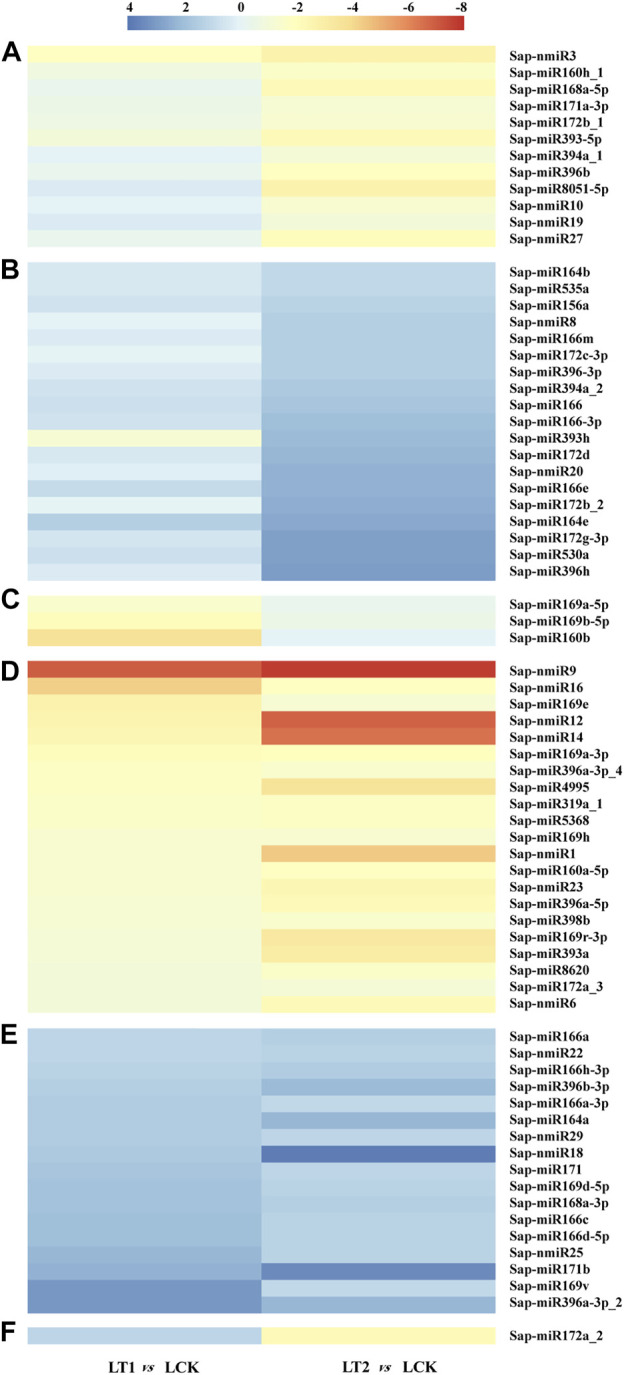
Differentially accumulated *S. apetala* miRNAs after 0 days (LCK), 1 day (LT1) and 28 days (LT2) of salt treatment. Relative abundance level was calculated with Log_2_(TPM_LT1_/TPM_LCK_) and Log_2_(TPM_LT2_/TPM_LCK_). These differentially accumulated miRNAs were divided into six clusters, represented by **(A–F)**.

Furthermore, these salt-regulated miRNAs could be divided into six categories on the basis of their accumulation patterns (Figures 5A–F). The miRNAs shown in Figures 5A,B did not exhibit significant changes in the accumulations at 1 day, but at 28 days, their modulation varied. Sap-miR169a-5p, Sap-miR169b-5p and Sap-miR160b demonstrated similar patterns of dynamic alterations during salt treatment, as their accumulations changed only after 1 day of salt treatment (Figure 5C). These miRNAs were up-regulated in LT1 vs*.* LCK and LT2 vs*.* LCK (Figure 5D). In contrast, a repression occurred both at 1 and 28 days in the other class (Figure 5E). In the last group, Sap-miR172a_2 showed induction at 1 day, but it was repressed at 28 days, which is unlike the pattern described above (Figure 5F). The various alterations of abundances for miRNAs implied that they played various roles under salt stress.

Predicting the targets of miRNAs would be essential for better understanding the functions of these salt-responsive miRNAs. A total of 300 genes were predicted as targets for 67 salt-responsive miRNAs (56 known and 11 novel), corresponding to 409 miRNA-target regulatory interactions for conserved and 62 pairs for novel miRNAs (Supplementary Datas S4, S5). We were unable to predict the targets of 6 miRNAs, possibly due to the insufficient *S. apetala* mRNA sequences. Among these expected targets, 25 (8.3%) targets have not been functionally annotated (Supplementary Data S5). The remaining annotated target genes participated in a broad spectrum of plant growth and development activities. Many of the predicted target genes were homologous to those encoding essential stress-related transcription factors (TFs), including MYB-domain transcription factor (MYB), APETALA2-like (AP2), homeodomain-leucine zipper transcription factor (HD-ZIP), nuclear transcription factor Y (NFYA), auxin response factor (ARF) family and WRKY transcription factor 22(WRKY22). Moreover, some target genes encoding enzymes or functional proteins that might be involved in plant metabolism, such as photosystem II PsbM protein, UDP-glucuronate decarboxylase (UXS1), protein phosphatase 2C (PP2C), and thioredoxin 1, were also identified. By annotation of targets, most transcripts (171 out of 300) were directly or indirectly implicated in plant salt stress responses, which had the largest proportion of signaling transduction (137, 80.1%), followed by morphological adaption (42, 24.6%), protein turnover (20, 11.7%), basic metabolism adaption (15, 8.8%), ion homeostasis (13, 7.6%), detoxification-related (5, 2.9%), osmotic protection (2, 1.2%) and nutrient modulation (1, 0.6%) (Supplementary Data S5). In general, these results implied that miRNAs might be involved in diverse biological processes under salt stress in *S. apetala*.

### Co-Expression and Regulation Analysis of *Sonneratia*-Specific MicroRNAs

We mined the alterations in miRNA abundances in other species that were subjected to the same period of salt treatment. A total of 10 miRNAs exhibit a difference when compared to their levels in the leaves of *S. apetala* and *Gossypium hirsutum* Linn. treated with salt for 1 day (Peng et al., 2014) (Supplementary Table S2). Out of these, six miRNAs showed inverse trends of accumulation in *S. apetala* and *G. hirsutum* under salt stress conditions. For instance, Sap-miR160a-5p and Sap-miR160b were down-regulated in *S. apetala*, but up-regulated in *G. hirsutum*, whereas Sap-miR166d-5p and Sap-miR396a-3p_2 were up-regulated in *S. apetala* but down-regulated in *G. hirsutum*. Additionally, Sap-miR168a-3p and Sap-miR169v were induced by salt in *S. apetala*, but were not significantly changed in response to salt treatment in *G. hirsutum*. Furthermore, 35 known miRNAs were considered to be *Sonneratia*-specific in genome organization. As showed in Supplementary Data S6, Sap-miR166a-3p targeted eight HD-ZIP proteins in *S. apetala* (Supplementary Data S6), but Homeobox-leucine zipper family protein (ATHB-15) was identified to be the target in *Populus euphratica* Oliv. (Li et al., 2013). Particularly, these 45 known miRNAs, of which 10 and 35 were specific in expression and genome organization, respectively, as well as the 17 differentially accumulated novel miRNAs were considered to be *Sonneratia*-specific.

**FIGURE 6:**
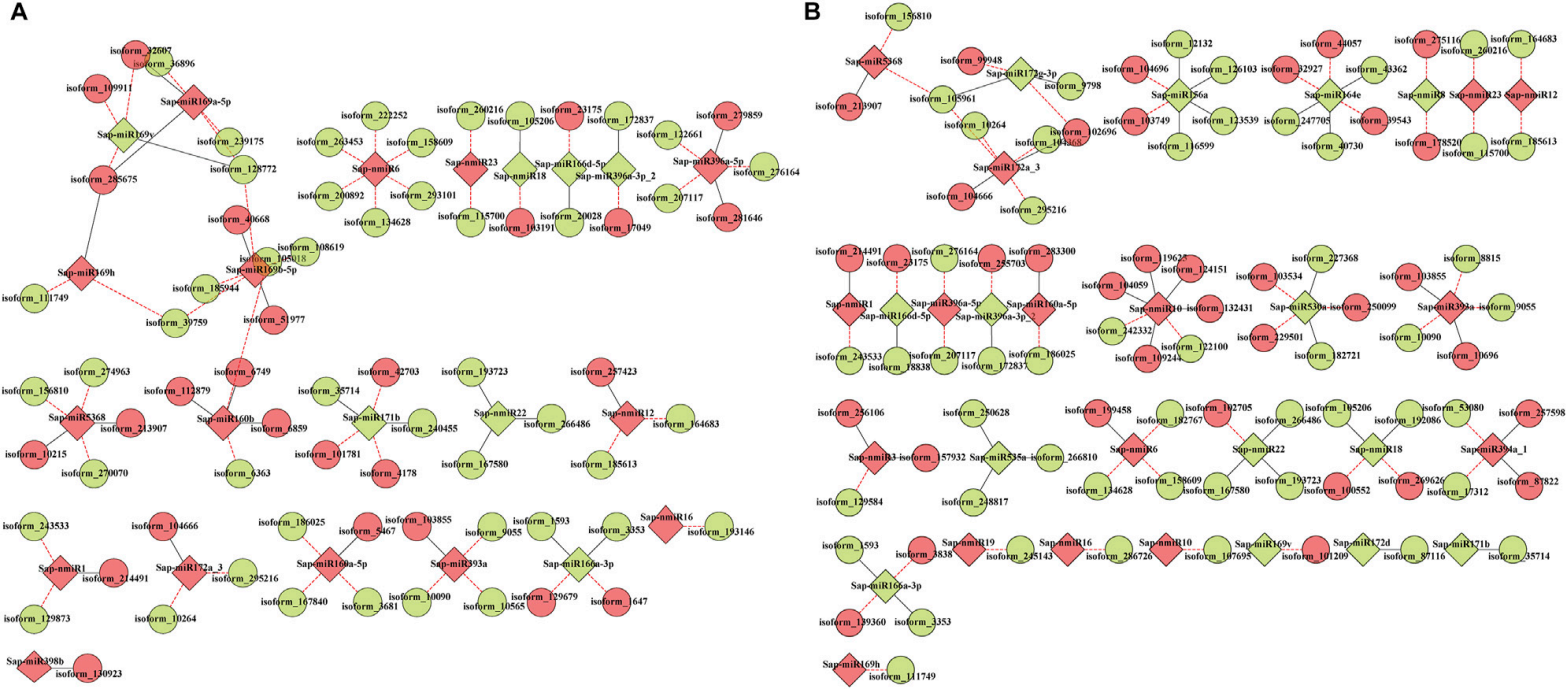
Co-expressed miRNA-target pairs of *Sonneratia*-specific in samples treated with salt for 1 day **(A)** and 28 days **(B)**. Solid lines represent positive correlations and dashed lines represent negative correlations. miRNAs and targets are marked by rhombus and circles, respectively; red/green shading represents down/up regulated miRNAs or target genes.

**FIGURE 7:**
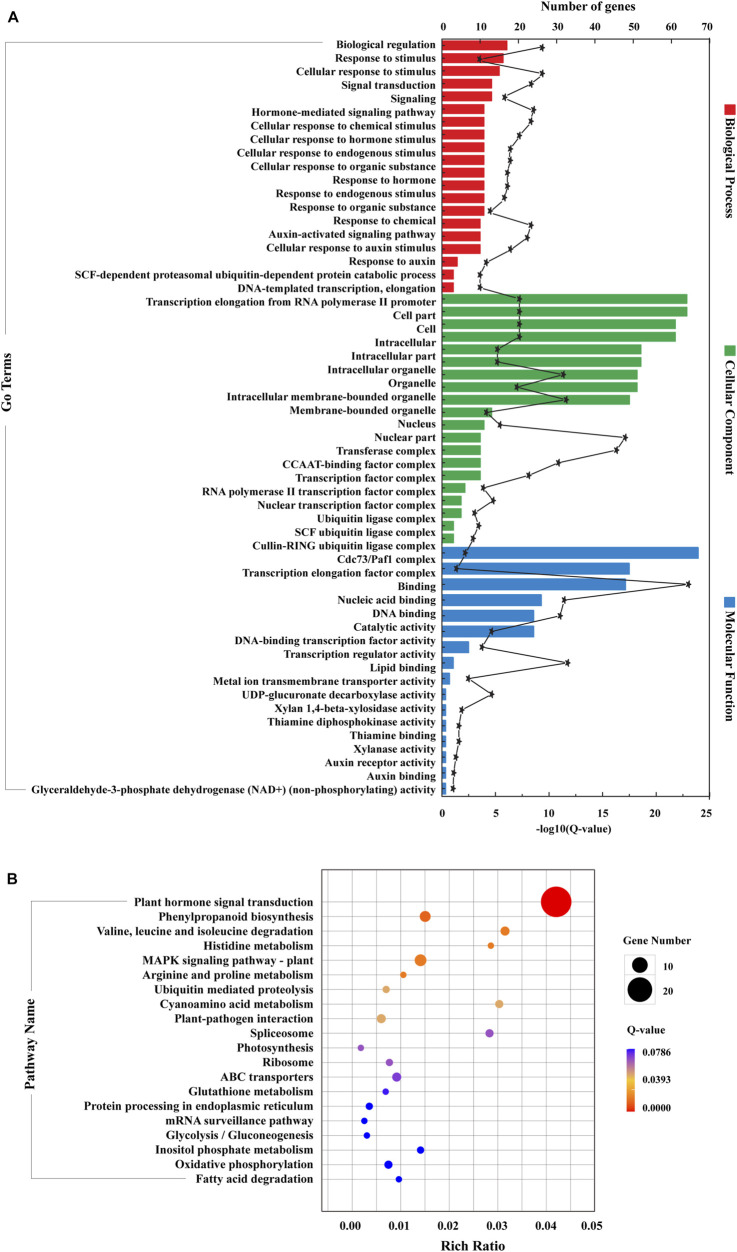
GO **(A)** and KEGG **(B)** classification of target genes that comprised the co-expressed miRNA-target regulatory network. For GO-based enrichment analysis, the 20 most abundant subcategories of each category (biological processes, cellular components and molecular functions) were listed and the first 20 significantly enriched pathways were presented for KEGG analysis. All the enrichment items were listed if the total was less than 20.

Subsequently, RNA-seq comparison of three groups (LCK, LT1, and LT2) for corresponding targets of the 62 *Sonneratia*-specific miRNAs were performed (Supplementary Data S7–S9), and differentially expressed target genes that exhibited significant expression correlations with their respective miRNAs (|r| > 0.75, *p* < 0.01, Q < 0.05) were selected for further exploration. To discover co-accumulated miRNA-target interactions for *Sonneratia-*specific miRNAs, we also focused on their alteration patterns upon salt stress. Particularly, miRNA-target pairs, of which miRNA and targes showed positive correlations and responded to stress in a congruous way were considered to be positively related, whereas targets exhibiting negative correlations and opposite tendency with their referred miRNAs were defined to be negative. Finally, 140 miRNA-target interactions were identified to be co-expressed after salt treatment, representing 34 *Sonneratia*-specific miRNAs and 131 target genes (Supplementary Datas S8–S10). To visualize the two-way interaction between miRNA and targets, an internal gene-gene network was constructed according to these miRNA-target pairs (Figure 6). Overall, 78 and 91 miRNA-target pairs showed negative or positive interactions independently at 1 and 28 days of salt stress whereas 29 exhibited regulatory relationships at both the time points. It was evident that one miRNA could be co-expressed with 1-8 target genes, among which the down-regulated Sap-miR396a-5p and Sap-nmiR10 could regulate five and seven mRNAs in LT1 vs*.* LCK and LT2 vs*.* LCK comparison, respectively. Simultaneously, Sap-miR169a-5p and Sap-miR169h could positively co-regulate isoform_285675 (NFYA, nuclear transcription factor Y) under early-(1 day) salt stress, while Sap-miR172a_3 and Sap-miR5368 exhibited negative correlations with their common target gene, isoform_105961 (AP2), after a salt-treatment for 28 days. To further probe the possible role of *Sonneratia-*specific miRNAs in response to salt stress, GO-based enrichment analysis was carried out on all the identified target genes in the regulatory interactions. The target genes were significantly mapped to 75 biological processes, 29 cellular components and 16 molecular functions (*p* < 0.05, Q < 0.1; Figure 7A; Supplementary Data S11). With respect to biological processes, the main terms were “biological regulation” (GO: 0065007, 17), “response to stimulus” (GO: 0050896, 16), and “cellular response to stimulus” (GO: 0051716, 15). The predominant terms implicated in cellular components were “cell part” (GO: 0044464, 64), “cell” (GO: 0005623, 64), “intracellular” (GO: 0005622, 61), and “intracellular part” (GO: 0044424, 61). For their molecular functions, the “binding” (GO: 0005488, 67) and “nucleic acid binding” (GO: 0003676, 49) were the most abundant subcategories. These classifications suggested that these miRNA targets, associated with salt tolerance, were primarily related to binding, stimulus, cellular metabolic, and other metabolic processes. When pathway analysis was performed by KEGG annotation, we were able to enrich 63 *Sonneratia-*specific miRNA targets to 34 pathways related to, for instance, metabolism of starch and sucrose (ko00500), spliceosome (ko03040), and fatty acid degradation (ko00071). (Figure 7B; Supplementary Data S12). The majority of representative pathways for the targets under the “metabolism” category were involved in the phenylpropanoid biosynthesis (ko00940; 6, 17.6%). Two signal transduction and one membrane transport pathways were classified into environmental information processing and thus were related to salt stress resistance. The only pathway categorized under the “organismal systems” (environmental adaptation) category was plant-pathogen interaction (ko04626; 4, 11.8%). The annotations of these target genes may provide new clues into the salt tolerance response in *S. apetala*.

**FIGURE 8:**
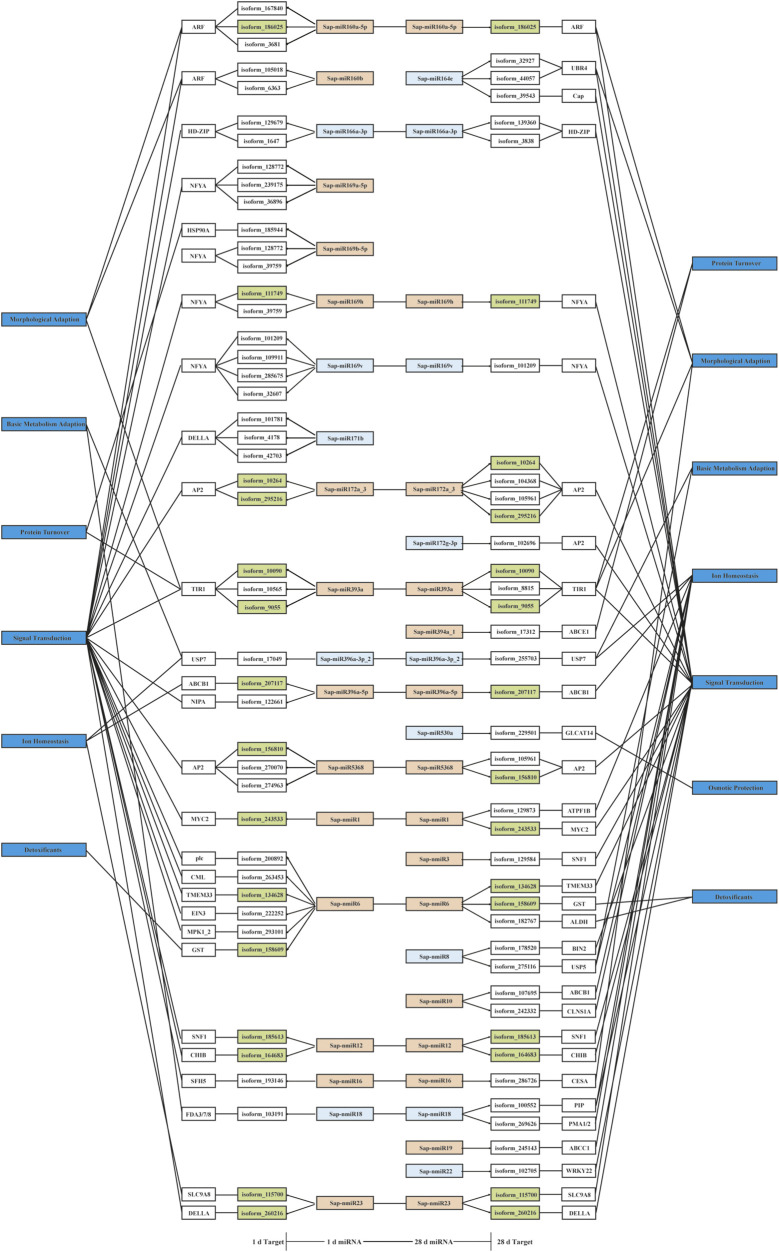
A proposed regulatory network of negatively related *Sonneratia*-specific miRNA-target pairs. The color of the miRNA box indicates the type of expression pattern: orange, down regulated; light blue, up-regulated. Green box indicates that targets were negatively regulated by the respective miRNAs at two time points (1 and 28 days) of salt treatment.

After investigating negatively related miRNA-target pairs, 82 pairwise interactions were selected for further analysis (Supplementary Data S10); of these, 17 exhibited negative regulatory relationship in both of the comparisons (LT1 vs*.* LCK and LT2 vs*.* LCK; Supplementary Table S3). *Basic endochitinase B* (*CHIB*) was inversely regulated (correlation coefficient, r = −0.86) by its respective miRNA. The level of *CHIB* transcripts was up-regulated in leaves treated with salt for 1 and 28 days, while a continuous decrease in Sap-nmiR12 levels was observed after both the salt treatments. Annotation of target genes indicated that a few transcripts were likely to participate in plant salt stress response (Figure 8). For instance, a Sap-nmiR6 target was GST, which was involved in “ROS scavenging protection” (Tian et al., 2019); the co-target gene of Sap-miR396a-5p and Sap-nmiR10, *ATP-binding cassette subfamily B member 1* (*ABCB1*), was related to “ion homeostasis” (Tian et al., 2019); Sap-miR160a-5p, Sap-miR160b, Sap-miR164e, Sap-miR393a, and Sap-nmiR16 might play roles in morphological adaption by targeting 2 *ARFs*, UBR4 (Feng et al., 2015), *TIR1* (McDiarmid et al., 2020), and *CESA* (Zaaba et al., 2021), respectively. It was noteworthy that the majority of the negative *Sonneratia*-specific miRNAs (77.4%) regulated the genes with function in stress signal perception and transduction, of which, 15 were predicted to target potential TF genes such as *MYC2*, *HD-ZIP*, and *NFYA*. These *Sonneratia*-specific miRNAs and their targets related to salt stress response allowed us to gain new insights into understanding stress tolerance mechanism in *S. apetala*.

## Discussion

### Small RNA Sequencing and Identification of MicroRNAs in *Sonneratia apetala*

Salt stress adversely influenced plant growth and development. To cope with salinity, plants adapt themselves through modulating numerous salt-responsive genes at the transcriptional and post-transcriptional levels. miRNAs are well known to function as ubiquitous regulators of gene expression and play crucial roles in a plant’s response to stress (Wani et al., 2020; Pagano et al., 2021). Increasing number of studies have demonstrated that miRNA-guided gene regulation plays a significant role during the salt stress response of various plant species (Xu et al., 2021). *S. apetala* is a pioneer mangrove species and is widely used for afforestation, which is important for keeping the ecological balance of a coastal zone. Studies of miRNAs related to stress adaptation of mangroves have only been reported in *A. marina* (Khraiwesh et al., 2013), *B. gymnorrhiza* and *K. candel* (Wen et al., 2016) for mangrove plants, and no study has been conducted on *S. apetala* for identification of salt-regulated miRNAs and their target genes.

In this study, more than 29 million sRNA reads were produced by deep sequencing for each library of *S. apetala* in the control and salinity treatments, imparting adequate sequencing depth for further analysis (Table 1). The sRNAs of *S. apetala* contained different types of RNAs, including miRNA, rRNA, snoRNA, snRNA, and tRNA (Table 2). Majority of the sRNA reads were unannotated, which possibly attributed to the scarcity of genome information on this species, and implied that numerous sRNAs still remain to be identified in *S. apetala*. The annotation performed here was in line with previous reports of other plants such as cotton (Zhan et al., 2021) and *Ipomoea batatas* L. (Yang et al., 2020). Analysis of the length distribution of these sRNA indicated that most were 21-nt and 24-nt (Figure 1), which has also been observed in other mangrove species, including *Bruguiera gymnorrhiza* and *Kandelia* (Wen et al., 2016). The maximum abundance of 21 nt sRNAs in all the libraries suggested that sRNAs with this length pattern might play more prevailing roles in the salt stress response of *S. apetala*. The separation in the length of sRNAs may have resulted from the enzymes that process it. For instance, sRNAs processed by DCL1, DCL2, and DCL3 in plants are 21, 22 and 24 nt-long, respectively (Necira et al., 2021).

According to miRNA prediction criteria, a total of 143 miRNAs, including 114 known and 29 novel miRNAs, were detected from the nine small RNA libraries (Supplementary Datas S1, S2). Most of the known miRNAs were 21 nt in length, and the novel miRNAs were mainly 23 and 24 nt (Figures 2B, 3B). The importance of length distribution for miRNA lies in the fact that it allows easy interaction with the AGO proteins: In general, the 21 or 22 nt miRNAs tend to function as silencing complexes with AGO1 proteins and cleave the target mRNAs, whereas 24 nt miRNAs guide DNA methylation by binding to AGO4 (Yang et al., 2020). Furthermore, it was inferred that the read copies of miRNAs ranging from zero to hundreds of thousands mirrored their diverse expression levels in *S. apetala*. For example, Sap-miR166a-3p, Sap-miR167d_1 and Sap-miR166d-5p had an extraordinarily high number of reads and averaged to 288,409, indicating that these miRNAs possibly accumulated at a higher level, whereas Sap-nmiR2 and Sap-nmiR19 showed low abundance of less than 100. Overall, the diversity of *S. apetala* miRNAs was also reflected in their highly variable abundances. Notably, known conserved miRNAs usually had relatively higher levels of accumulation when compared with those newly identified (Supplementary Datas S1, S2). This was a common characteristic of plant miRNAs and supported by previous studies (Feng et al., 2015). Additionally, a striking divergence also existed in the accumulation patterns of miRNAs at different time points of salt treatment as both, congruously and oppositely regulated miRNAs were detected (Figure 4; Supplementary Datas S1, S2). Our findings provide a strong basis for further in-depth miRNA studies in *S. apetala*.

### Characterization of Salt Stress-Responsive MicroRNAs and Their Targets in *Sonneratia apetala*

Identification of differentially expressed miRNAs and their subsequent functional dissection would aid in the understanding of the response mechanism in *S. apetala* under salt stress. It is well known that salt adaptation is a long-term and dynamic process that involves many morphological, physiological, molecular, and cellular processes (Feng et al., 2020). To probe the dynamic changes of miRNAs during the saline stress, we compared their accumulation from samples treated with salt for 1 and 28 days to that of control libraries (0 days). totally, 73 miRNAs (56 known miRNAs and 17 novel) were found to be regulated by salinity (*p* < 0.01, Q < 0.05; Figure 5; Supplementary Data S3). Earlier studies have demonstrated that miR156, miR159, miR160, miR168, miR169, miR171, miR172, miR393, and miR396 were the major salt stress-regulated miRNAs in plants (Alzahrani et al., 2019). All of these were also identified in our study, indicating the existence of common salt stress-related miRNAs. To the best of our knowledge, some conserved miRNAs were found to be regulated by salt stress for the first time in this study. This includes Sap-miR394a_1 and Sap-miR8051-5p, which maintained a relatively constant level at 1 d of stress but were significantly inhibited at 28 days. Additionally, a few novel salt-responsive miRNAs were identified in *S. apetala* (Supplementary Data S3). Several miRNAs, such as Sap-miR395p-3p, Sap-nmiR9 and Sap-nmiR15, were detected in certain specific libraries, indicating their thorough induction or repression under salt stress. Previous studies have reported that miRNA168 in *Arabidopsis* and maize was coordinately accumulated by salinity, while maize miR167 and *S. linnaeanum*-miR399b were decreased (Yang et al., 2020). Additionally, we noted that some stress-regulated miRNAs, identified in this study, might be fine-tuned across adaptive responses to various stresses. For example, miR169 and miR319 were considered as a bridge, linking plant responses to ABA, drought and salt stress (De la Rosa et al., 2019; Salgado et al., 2021); miR398 was reported to play vital roles in various stresses such as water deficit (Yang et al., 2020), oxidative stress (Li et al., 2020), nutrient deficiency (Islam et al., 2022), salt stress (He et al., 2021), and bacterial infection (Salamon et al., 2021). This was probably due to the shared regulatory genes that are modulated by these miRNAs across distinct stress responses, attempting to reveal complex miRNA-mediated gene regulation was involved in the cross-response to abiotic and biotic stresses. For a further in-depth study of miRNA-mediated stress adaptation in mangroves, we would establish more comparisons to uncover crucial miRNAs that respond to stress. Particularly, miRNA profiles from salinity-exposed libraries would be compared to those of control samples growing for the same period of time.

A crucial step in understanding the potential regulatory roles of these salt-responsive miRNAs is the prediction and analysis of regulation of their targets. A total of 300 target genes for 67 significantly differentially accumulated miRNAs were identified in this study (Supplementary Datas S4, S5). The response to salt stress in plants is coupled with a wide range of intracellular processes, including signal sensing and transduction, transcription reprogramming, protein biosynthesis regulation that finally ascertain physiological changes to cope with stress. TFs play prominent roles during acclimation response when plants are subjected to severe environments. Here, 29.3% (88) of the 300 target genes encoded for TF family members, including MYB, AP2, HD-ZIP, NFYA, ARF and WRKY (Supplementary Datas S4, S5), which were reported to activate stress-related genes (Baillo et al., 2019; Yoon et al., 2020). Many other target genes encode for enzymes or functional proteins are considered to participate in salt stress response process. In *B. gymnorhiza* and *R. mangle* mangroves, miR172, miR394, and miR396 have been identified to act as important regulators of salt stress adaption via the regulation of stress signaling, oxidative resistance, and defense responses by targeting P-loop containing nucleoside triphosphate hydrolase superfamily protein (NTPase), UDP-glycosyltransferase (UGT) and Rhodanese/Cell cycle control phosphatase superfamily protein (RHOD), respectively (Wen et al., 2016). Herein, *Sonneratia*-specific targets of these miRNAs, involved in stress tolerance, were identified to participate in multiple processes of saline responses (Supplementary Data S5). Some targets encoded formetabolic enzymes. Sap-nmiR1 and Sap-nmiR18 could commonly target an *acyl-lipid omega-3 desaturase* (*FDA*). The antisense expression of *Arabidopsis FDA7* gene could reduce tobacco resistance to salinity (Xu et al., 2019). A gene encoding for a ubiquitin-specific protease (UBP) implicated in sugar metabolism (Feng et al., 2015), was the shared target of Sap-miR396a-3p_2 and Sap-nmiR8. Additionally, UBPs could also functions as Na^+^/H^+^ antiporter regulators, modulating monovalent cations and subsequent pH homeostasis in *Arabidopsis* (Zhou H. et al., 2012). Therefore, Sap-miR396a-3p_2/Sap-nmiR8-targeting *UBP*s may exhibit critical functions in maintenance of Na^+^ and K^+^ homeostasis in *S. apetala*, which is essential for plant survival during exposure to saline stress. Inclusively, Sap-nmiR6 might also play important roles in ion homeostasis by regulating the expression of *nitrate transporter* (*NRT*) under salt conditions. As an essential nutrient required for plant growth, nitrate serve as a signal that modulates plant development, and nitrate uptake is stimulated by salinity (Sa et al., 2019). The uptake and distribution of nitrate at the whole plant level are determined by joint activity of nitrate transporters. Oxidative stress is regarded as the most severe form of salt stress, which results from excessive accumulation of reactive oxygen species (ROS). Herein, several target genes involved in oxidative resistance were found to be cross regulated by miRNAs under saline conditions. Sap-miR4995 is predicted to target *L-ascorbate oxidase homolog* (*AOXH*) and *thioredoxin 1* (*trxA*); these proteins act as significant ROS scavenging enzymes and are involved in modifying intracellular ROS expression levels (Vishwakarma et al., 2015; González et al., 2020). Also, Cu/Zn superoxide dismutase, encoded by *Cu/Zn-SOD*, is widely reported to function in scavenging excess ROS in plants exposed to salinity (Sarkar et al., 2022) and here is predicted to be Sap-miR4995/Sap-nmiR18-regulated. Notably, *Cu/Zn SODs* were identified as targets of miR398 in *B. gymnorhiza* and *A. marina* (Khraiwesh et al., 2013; Wen et al., 2016). The detoxificant enzyme gene, *aldehyde dehydrogenase* (*ALDHs*) was targeted by a novel miRNA (Sap-nmiR6) in *S. apetala*, but by miR399 in *A. marina* (Khraiwesh et al., 2013). These observations indicate a difference in miRNA-mediated salt stress response for different mangrove plants. These miRNA-target pairs may serve as a supplementary resource for translating miRNA-mediated gene regulation for enhancing plant stress tolerance.

### Mediation of a Potential Co-Expression Network *via Sonneratia*-Specific MicroRNAs in Response to Salt Stress

Although a large number of stress-related miRNAs were evolutionarily conserved in plants, some miRNAs have variable regulatory patterns across different species. According to the genome and abundance characteristic of salt-responsive miRNAs identified in this study, 62 (45 known and 17 novel) miRNAs potentially modify the acclimation response to salt stress in a species-specific manner (Supplementary Table S2; Supplementary Data S2, S6). After combining correlation coefficient and accumulation patterns of these *Sonneratia*-specific miRNAs and their target counterparts under salt stress, 34 miRNAs and 131 targets producing 140 miRNA-target interactions were determined to be co-expressed after salt treatment (Figure 6; Supplementary Data S8–S10). Within this co-expression network, the number of pairwise miRNA-target interactions differed dramatically at two-time points of salt treatment. In LT1 vs*.* LCK comparison, 78 miRNAs-target pairs (29 positive and 49 negative) were significantly induced under salt stress (Figure 6A), whereas 91 miRNAs-target interactions (42 positive and 49 negative) were determined in LT2 vs*.* LCK comparison (Figure 6B). These findings implied that miRNAs, which exhibited concordant positive or negative regulatory relationships with their corresponding targets throughout the time-course of salinity, may have similar response mechanisms in *S. apetala*. Further analysis demonstrated that 111 miRNAs displayed disparate changes in their accumulation in *S. apetala* under early-(1 day) and late-stages (28 days) of salt stress (Supplementary Data S10). This differential pattern of miRNA accumulation may be liable to a distinct salt response mechanism at the two-time points. GO enrichment analysis of their putative targets contained many abiotic stress-related categories, such as “response to stimulus”, “intracellular” and “nucleic acid binding” (Figure 7A). According to KEGG pathways analysis, all the signal transduction pathways were classified into environmental information processing (Supplementary Data S12). It is possible that these signal transduction pathways may promote high salt tolerance of *S. apetala*. Further, phenylpropanoid biosynthesis, starch and sucrose metabolism, fatty acid degradation, spliceosome, and other pathways were activated *via* various enzymes under salt stress, which may initiate the production of salt-induced signaling molecules, ion channels, oxidation protectants, ROS, and other stress-related metabolites (Liu et al., 2020).

Generally, miRNAs modulate the specific genes by pairing with mRNAs transcripts, leading to degradation or translation inhibition and therefore their expression profiles should be negatively related (Tyagi et al., 2019). There is no doubt that the negatively correlated miRNA-target pairs should be emphasized. On this basis, we separated out 82 pairwise miRNA-target interactions that exhibited negative correlations for further analysis (Supplementary Data S10). Among these, two of the novel miRNAs, Sap-nmiR10 and Sap-nmiR23, exhibited cross-functionality with the known Sap-miR396a-5p and Sap-miR171b for the targets, *ABCB1* and *DELLA*, respectively (Figure 8). This indicated that new *Sonneratia*-specific miRNAs could evolve to co-target certain genes with conserved miRNAs and shared common response mechanisms under salt stress. Particularly, 13 miRNAs and their 18 target counterparts (Supplementary Table S4) were categorized into the “environmental information processing” category, which were involved in plant hormone signal transduction (ko04075), MAPK signaling pathway–plant (ko04016) and ABC transporters (ko02010). Acclimation response of plants to salt stress requires coordination and integration of multiple phytohormones, including auxin (IAA) (Ribba et al., 2020), ethylene (Wang and Huang, 2019), ABA (Zhu, 2016), and jasmonic acid (JA) (Ali and Baek, 2020). Previous studies have demonstrated that ethylene-induced the detoxification machinery and increased plant salt tolerance (Zhao et al., 2020). As a TF that mediates core ethylene signaling, ethylene-insensitive protein 3 (EIN3) was stabilized by salinity and promoted plant survival to saline stress via the DELLA proteins (Zhao et al., 2020), which was targeted by Sap-miR171b and Sap-nmiR23. Also, auxin participates in multiple processes of salt stress responses, modulating a complex balance of biosynthesis, signaling, ion transport, and finally fine-tuning of physiological changes (Ribba et al., 2020). Notably, auxin response factors (ARFs) were reported to serve as a point of crosstalk between the two hormones, ethylene and IAA, which not only function in auxin signaling, but also play a vital role in ethylene responses (Ribba et al., 2020). In this study, both Sap-miR160b and Sap-miR160a-5p were down-regulated at the early-stage (1 day) of salt stress, resulting in an increased expression of the target *ARF* (Supplementary Data S10). This increase in *ARF* expression may serve crucial functions in salt resistance of *S. apetala*. Sap-miR393a-targeted *TIR1*, encoding an F-box family protein, is a negative regulator in auxin signaling (Fendrych et al., 2018), which was also implicated in ARF-medicated signal transduction. Two out of three target genes, isoform_9055 and isoform_10090, encoding TIR1, exhibited an increased expression until the end of the salt treatment. The accumulation of their miRNA regulator, Sap-miR393a, was down-regulated after 1 and 28 days of salt treatment (Supplementary Data S10), indicating a pivotal role of Sap-miR393a-*TIR1* interaction in salt tolerance of *S. apetala.* Further, analysis of transgenic *Arabidopsis* and rice plants had revealed that the overexpression of OsmiR393 led to enhanced sensitivity to salinity and alkaline stresses (Giri et al., 2021). In light of the above observations, we inferred that miR393 was a negative modulator of plant salt tolerance. MAPK cascades are important signaling pathways related to plant responses to salt stress (Kumar et al., 2020). In this study, four target genes, *MYC2*, *WRKY22*, *CHIB* and *MPK1_2*, which were paired by Sap-nmiR1, Sap-nmiR22, Sap-nmiR12 and Sap-nmiR6, respectively, were identified to be associated with MAPK pathway in our KEGG annotation (Supplementary Table S4). These results indicated a crucial role for novel miRNAs during the *S. apetala* survival under saline conditions. Further, ABC transporters are known to mediate a H^+^-Na^+^-Cl^−^ symport reaction and can improve salt-adapted cells survival during downshifts of extracellular ion concentration (Velamakanni et al., 2009). The co-target of Sap-miR396a-5p and Sap-nmiR10, an *ABCB1,* and Sap-nmiR19-targeted *ABCC1* were induced under salt conditions, which indicated the importance of ABC transporters in ion homeostasis and compatible molecule accumulation during the process of saline tolerance in *S. apetala*. Additionally, target genes involved in basic metabolism of sugar, lipid and amino acid, detoxification, osmotic protection, protein turnover, as well as morphological adaption were also identified in the negatively correlated miRNA-target pairs (Figure 8). The discovery of many salt-regulated miRNA indicates that a complicated regulatory network is implicated in response to salinity in *S. apetala*. Further efforts are needed to functionally confirm these putative interactions and probe the regulatory mechanism underlying these interactions in *S. apetala* adaptive response to saline conditions.

## Data Availability

The original contributions presented in the study are publicly available. These data can be found at: https://ngdc.cncb.ac.cn/gsa/, CRA006866 and CRA006863.
