# Supplementary material for: Integrated Full-Length Transcriptome and MicroRNA Sequencing Approaches Provide Insights Into Salt Tolerance in Mangrove (Sonneratia apetala Buch.-Ham.)
Source: Front Genet. 2022 Jul 11;13:932832. doi: 10.3389/fgene.2022.932832 (PMC9310009; doi:10.3389/fgene.2022.932832)
Supplement: Supplementary file 1 [file DataSheet1.PDF]

Supplementary Material

The following Supporting Information is available for this article:

Supplementary Figure S1 Pearson correlation analysis across samples based on gene expression patterns
Supplementary Figure S2 Pearson correlation analysis across samples based on gene expression patterns

Supplementary Table S1 Primer sequences used for RT-qPCR
Supplementary Table S2 *Sonneratia*-specific miRNAs with discrepant expression patterns at the same period of salt treatment
Supplementary Table S3 Negatively related miRNA-target pairs at both of the two time points (1 d and 28 d) of salt treatment
Supplementary Table S4 miRNA-target pairs with negative correlations that were categorized into the "environmental information processing" by KEGG analysis

Supplementary Data S1-S12 were provided in other formats (Excel), which were submitted as other separate files

1.1 Supplementary Figures

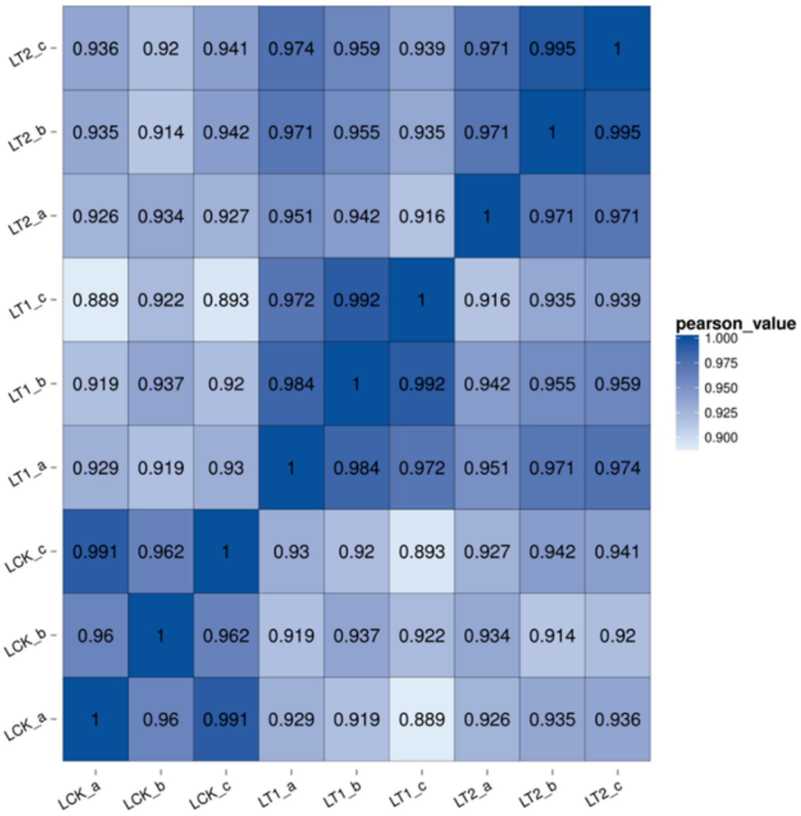

Supplementary Figure S1 Pearson correlation analysis across samples based on gene expression patterns. LCK, LT1 and LT2 represent samples treated with salt for 0 d, 1 d and 28 d, respectively; a, b, c represent the three repetitions.

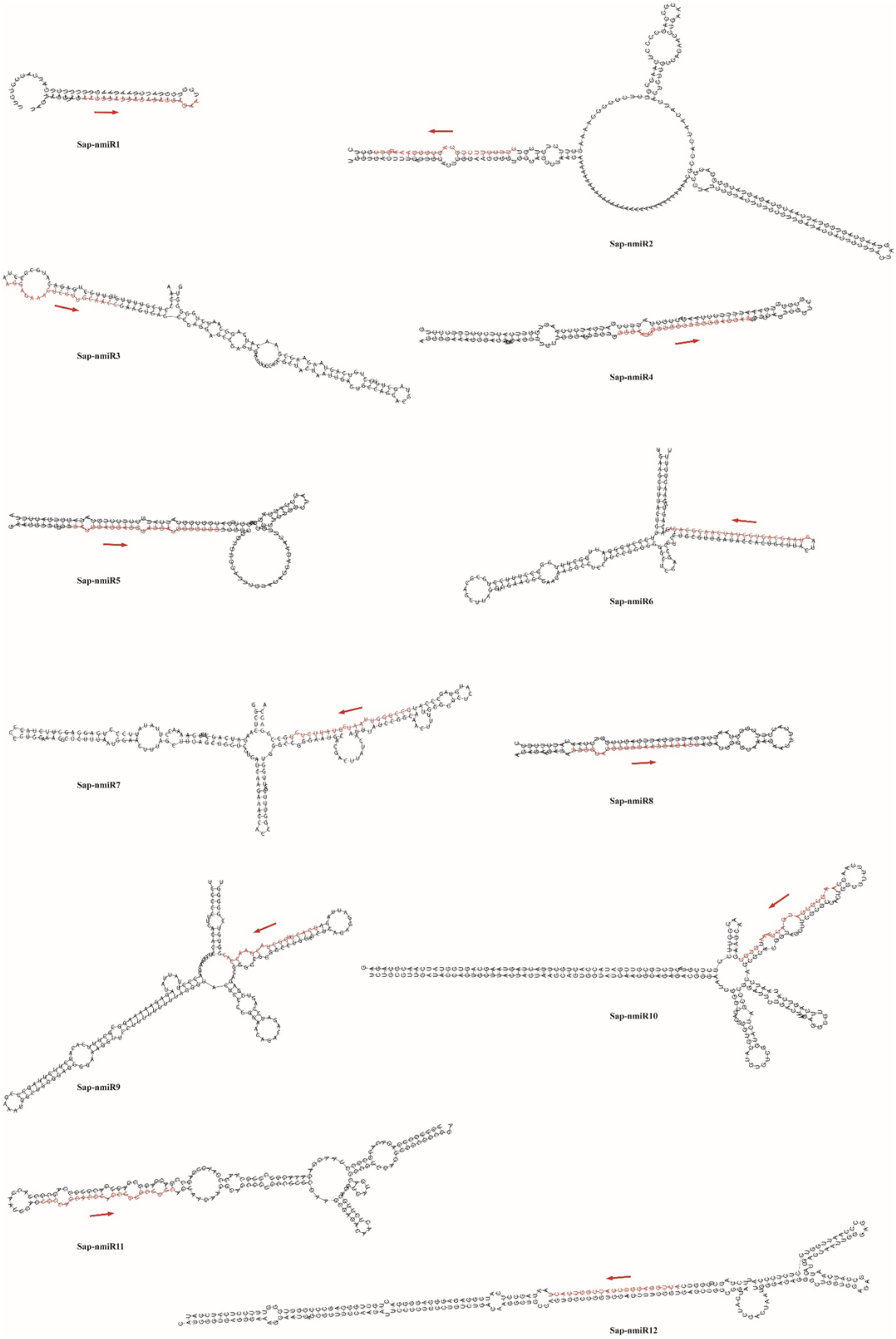
Sap-nmiR1
Sap-nmiR2
Sap-nmiR3
Sap-nmiR4
Sap-nmiR5
Sap-nmiR6
Sap-nmiR7
Sap-nmiR8
Sap-nmiR9
Sap-nmiR10
Sap-nmiR11
Sap-nmiR12

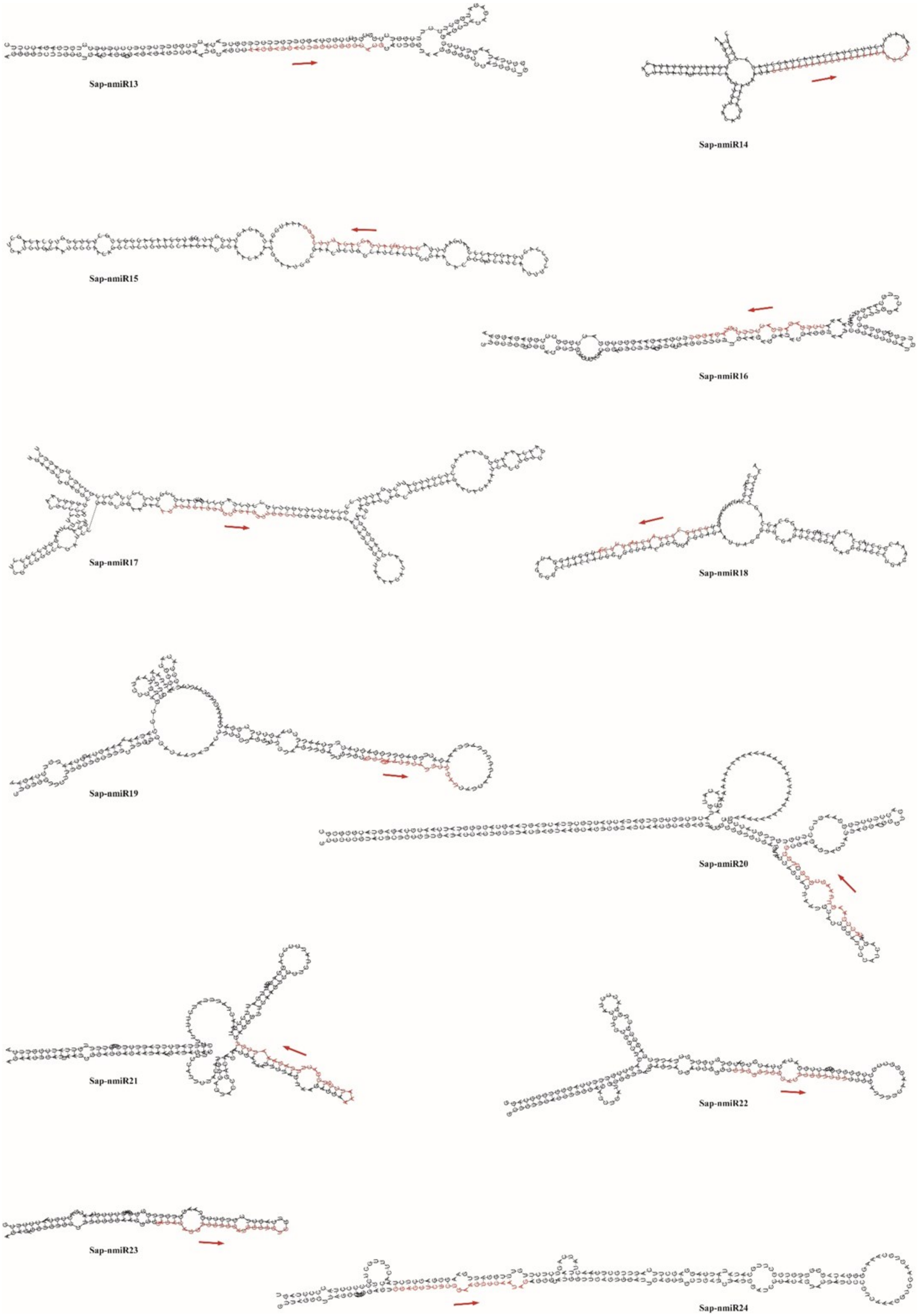
Sap-nmiR13
Sap-nmiR14
Sap-nmiR15
Sap-nmiR16
Sap-nmiR17
Sap-nmiR18
Sap-nmiR19
Sap-nmiR20
Sap-nmiR21
Sap-nmiR22
Sap-nmiR23
Sap-nmiR24

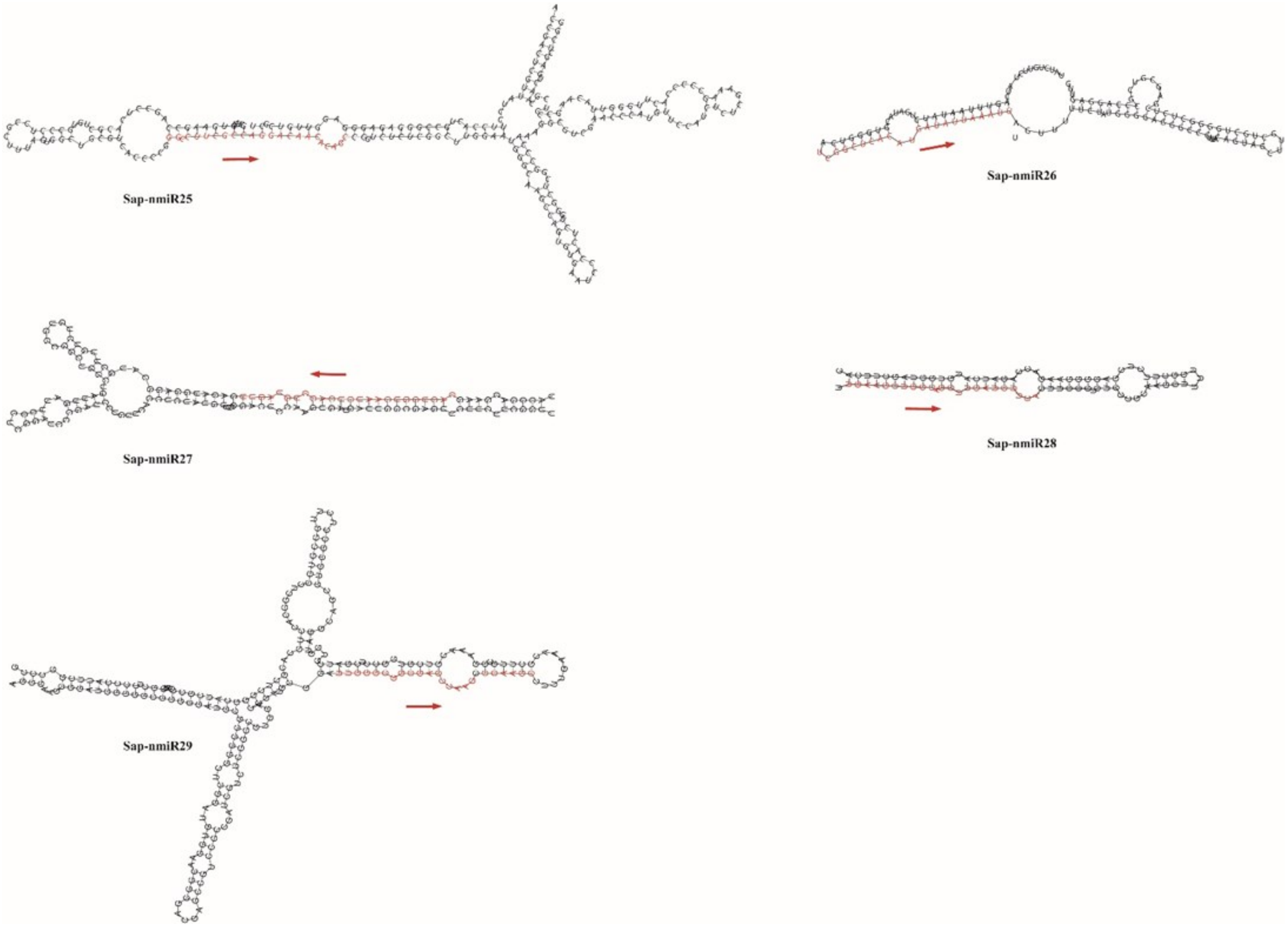

Supplementary Figure S2 Pearson correlation analysis across samples based on gene expression patterns. LCK, LT1 and LT2 represent samples treated with salt for 0 d, 1 d and 28 d, respectively; a, b, c represent the three repetitions.

1.2 Supplementary tables

Supplementary Table S1 Primer sequences used for RT-qPCR

**miRNA**	**Primer sequence (5′ →3′)**
Sap-miR166a-3p	TCGGACCAGGCTTCATTCCCC
Sap-miR168a-3p	CCCGCCTTGCATCAACTGAAT
Sap-nmiR22	GTGGGTGGCACTCGGTCCT
Sap-miR396-3p	AAGCTCAAGAAAGCTGTGGGA
Sap-nmiR16	CCTGAGATGACCTTCCGTAGAGGT
Sap-nmiR17	ATTGTCAGGTGGGGAGTTTGGCT
Sap-miR169d-5p	TAGCCAAGGATGACTTGCCT
Sap-nmiR6	GTAAGCAGTGGTATCAACGCAGA
Sap-nmiR28	TGAAGCTGCCAGCTTGATCTTCA
Sap-miR396b-3p	GCTCAAGAAAGCTGTGGGAAA
U6 (Forward primer)	CCTTCGGGGACATCCGATAAAA
U6 (Reverse primer)	GCAGGGGCCATGCTAATCTTCT
Universal reverse primer	GTGCAGGGTCCGAGGT

Supplementary Table S2 ***Sonneratia*****-specific miRNAs with discrepant expression patterns at the same period of salt treatment**

Reference miRNA	Species 1	miRNA ID	Up/Down-Regulation_1 d/0 d	Species 2	miRNA ID	Up/Down-Regulation_1 d/0 d
bna-miR160a, cme-miR160a, mdm-miR160a, mtr-miR160a, nta-miR160a, pab-miR160a, ppt-miR160a, ptc-miR160a, rco-miR160a, sbi-miR160a sly-miR160a, smo-miR160a	*Sonneratia apetala* Buch.-Ham.	Sap-miR160a-5p	Down	*Gossypium hirsutum Linn.*	bna-miR160a, cme-miR160a, mdm-miR160a, mtr-miR160a, nta-miR160a, pab-miR160a, ppt-miR160a, ptc-miR160a, rco-miR160a, sbi-miR160a, sly-miR160a, smo-miR160a	UP
gma-miR160b	*Sonneratia apetala* Buch.-Ham.	Sap-miR160b	Down	*Gossypium hirsutum Linn.*	gma-miR160b	UP
osa-miR166d-5p, aly-miR166d-5p	*Sonneratia apetala* Buch.-Ham.	Sap-miR166d-5p	Up	*Gossypium hirsutum Linn.*	osa-miR166d-5p, aly-miR166d-5p	Down
ptc-miR168a-3p, aly-miR168a-3p	*Sonneratia apetala* Buch.-Ham.	Sap-miR168a-3p	Up	*Gossypium hirsutum Linn.*	ptc-miR168a-3p, aly-miR168a-3p	-

gma-miR169v	*Sonneratia apetala* Buch.-Ham.	Sap-miR169v	Up	*Gossypium hirsutum Linn.*	gma-miR169v	-
ath-miR319a, rco-miR319a, rco-miR319c, vun-miR319a, nta-miR319a, mdm-miR319a	*Sonneratia apetala* Buch.-Ham.	Sap-miR319a_1	Down	*Gossypium hirsutum Linn.*	ath-miR319a, rco-miR319a, rco-miR319c, vun-miR319a, nta-miR319a, mdm-miR319a	-
gma-miR396a-3p	*Sonneratia apetala* Buch.-Ham.	Sap-miR396a-3p_2	UP	*Gossypium hirsutum Linn.*	gma-miR396a-3p	Down
aqc-miR396a, bcy-miR396a, bgy-miR396a, ghr-miR396a, nta-miR396a, ptc-miR396a, sbi-miR396a, tcc-miR396a	*Sonneratia apetala* Buch.-Ham.	Sap-miR396a-5p	Down	*Gossypium hirsutum Linn.*	aqc-miR396a, bcy-miR396a, bgy-miR396a, ghr-miR396a, nta-miR396a, ptc-miR396a, sbi-miR396a, tcc-miR396a	UP
osa-miR398b, ptc-miR398b, mtr-miR398b, vvi-miR398b, aqc-miR398b, rco-miR398b, tcc-miR398a, mdm-	*Sonneratia apetala* Buch.-Ham.	Sap-miR398b	Down	*Gossypium hirsutum Linn.*	osa-miR398b, ptc-miR398b, mtr-miR398b, vvi-miR398b, aqc-miR398b, rco-miR398b, tcc-miR398a,	Up

miR398b, cme-miR398a					mdm-miR398b, cme-miR398a	
gma-miR5368	*Sonneratia apetala* Buch.-Ham.	Sap-miR5368	Down	*Gossypium hirsutum Linn.*	gma-miR5368	-

Supplementary Table S3 Negatively related miRNA-target pairs at both of the two time points (1 d and 28 d) of salt treatment

miRNA	Target	Abbreviation of gene	Correlation Coefficient (pearson)	miRNA Up/Down-Regulation_0 d *vs* 1 d	Target Up/Down-Regulation_0 d *vs* 1 d	Co-expression Pattern_0 d *vs* 1 d	miRNA Up/Down-Regulation_0 d *vs* 28 d	Target Up/Down-Regulation_0 d *vs* 28 d	Co-expression Pattern_0 d *vs* 28 d
Sap-miR393a	isoform_10090	TIR1	-0.978262	down	up	negative	down	up	negative
Sap-miR166d-5p	isoform_23175	K22395	-0.933438	up	down	negative	up	down	negative
Sap-miR160a-5p	isoform_186025	K14486, ARF	-0.927046	down	up	negative	down	up	negative
Sap-nmiR23	isoform_115700	SLC9A8, NHE8	-0.914502	down	up	negative	down	up	negative
Sap-miR172a_3	isoform_295216	AP2	-0.884771	down	up	negative	down	up	negative
Sap-nmiR6	isoform_134628	TMEM33	-0.880796	down	up	negative	down	up	negative
Sap-miR169h	isoform_111749	NFYA, HAP2	-0.879197	down	up	negative	down	up	negative
Sap-nmiR12	isoform_164683	CHIB	-0.863965	down	up	negative	down	up	negative
Sap-nmiR1	isoform_243533	MYC2	-0.854079	down	up	negative	down	up	negative
Sap-nmiR6	isoform_158609	GST, gst	-0.852538	down	up	negative	down	up	negative
Sap-miR396a-5p	isoform_276164	CDL15	-0.84086	down	up	negative	down	up	negative
Sap-miR393a	isoform_9055	TIR1	-0.840273	down	up	negative	down	up	negative

Sap-miR5368	isoform_156810	AP2	-0.789513	down	up	negative	down	up	negative
Sap-nmiR23	isoform_260216	DELLA	-0.784088	down	up	negative	down	up	negative
Sap-miR172a_3	isoform_10264	AP2	-0.778621	down	up	negative	down	up	negative
Sap-nmiR12	isoform_185613	SNF1	-0.778319	down	up	negative	down	up	negative
Sap-miR396a-5p	isoform_207117	ABCB1, CD243	-0.752275	down	up	negative	down	up	negative

Supplementary Table S4 miRNA-target pairs with negative correlations that were categorized into the "environmental information processing" by KEGG analysis

Pathway ID	Pathway Name	Level 1	Level 2	Target gene	Salt-related pathway	miRNA	Abbreviation of gene	Annotated description
ko04075	Plant hormone signal transduction	Environmental Information Processing	Signal transduction	isoform_10090	protein turnover, morphological adaption, signaling transduction (ABA/IAA signaling)	Sap-miR393a	TIR1	transport inhibitor response 1
				isoform_9055	protein turnover, morphological adaption, signaling transduction (ABA/IAA signaling)	Sap-miR393a	TIR1	transport inhibitor response 1
				isoform_10565	protein turnover, morphological adaption, signaling transduction (ABA/IAA signaling)	Sap-miR393a	TIR1	transport inhibitor response 1
				isoform_105018	morphological adaption, signaling transduction (ethylene/IAA signaling)	Sap-miR160b	K14486, ARF	auxin response factor
				isoform_167840	morphological adaption, signaling transduction (ethylene/IAA signaling)	Sap-miR160a-5p	K14486, ARF	auxin response factor
				isoform_186025	morphological adaption, signaling transduction (ethylene/IAA signaling)	Sap-miR160a-5p	K14486, ARF	auxin response factor

				isoform_6363	morphological adaption, signaling transduction (ethylene/IAA signaling)	Sap-miR160b	K14486, ARF	auxin response factor
				isoform_260216	signaling transduction (ethylene signaling)	Sap-miR171b	DELLA	DELLA protein
				isoform_42703	signaling transduction (ethylene signaling)	Sap-miR171b	DELLA	DELLA protein
				isoform_101781	signaling transduction (ethylene signaling)	Sap-miR171b	DELLA	DELLA protein
				isoform_178520	signaling transduction (ABA signaling)	Sap-nmiR8	BIN2	protein brassinosteroid insensitive 2
				isoform_243533	signaling transduction (jasmonate signaling, MAPK signaling)	Sap-nmiR1	MYC2	transcription factor MYC2
				isoform_243533	signaling transduction (jasmonate signaling, MAPK signaling)	Sap-nmiR1	MYC2	transcription factor MYC2
ko04016	MAPK signaling pathway - plant	Environmental Information Processing	Signal transduction	isoform_102705	signaling transduction (MAPK signaling)	Sap-nmiR22	WRKY22	WRKY transcription factor 22
				isoform_164683	antioxidation, signaling transduction (MAPK signaling, hormone signaling)	Sap-nmiR12	CHIB	basic endochitinase B

				isoform_293101	signaling transduction (MAPK signaling)	Sap-nmiR6	MPK1_2	mitogen-activated protein kinase 1/2
				isoform_107695	ion homeostasis	Sap-nmiR10	ABCB1, CD243	ATP-binding cassette, subfamily B (MDR/TAP), member 1
ko02010	ABC transporters	Environmental Information Processing	Membrane transport	isoform_207117	ion homeostasis	Sap-miR396a-5p	ABCB1, CD243	ATP-binding cassette, subfamily B (MDR/TAP), member 1
				isoform_245143	ion homeostasis	Sap-nmiR19	ABCC1	ATP-binding cassette, subfamily C (CFTR/MRP), member 1
